# Empathy and Schadenfreude in Human–Robot Teams

**DOI:** 10.5334/joc.177

**Published:** 2021-08-05

**Authors:** Dorina de Jong, Ruud Hortensius, Te-Yi Hsieh, Emily S. Cross

**Affiliations:** 1 Institute of Neuroscience and Psychology, School of Psychology, University of Glasgow, Scotland, UK; 2 Istituto Italiano di Tecnologia, Center for Translational Neurophysiology of Speech and Communication, (CTNSC), Ferrara, Italy; 3 Università di Ferrara, Dipartimento di Scienze Biomediche e Chirurgico Specialistiche, Ferrara, Italy; 4 Department of Psychology, Utrecht University, Heidelberglaan 1, 3584 CS, Utrecht, The Netherlands; 5 Department of Cognitive Science, Macquarie University, 16 University Ave, Sydney, NSW 2109, Australia

**Keywords:** empathy, schadenfreude, intergroup bias, human-robot interactions, collaboration, social cognition

## Abstract

Intergroup dynamics shape the ways in which we interact with other people. We feel more empathy towards ingroup members compared to outgroup members, and can even feel pleasure when an outgroup member experiences misfortune, known as schadenfreude. Here, we test the extent to which these intergroup biases emerge during interactions with robots. We measured trial-by-trial fluctuations in emotional reactivity to the outcome of a competitive reaction time game to assess both empathy and schadenfreude in arbitrary human-human and human-robot teams. Across four experiments (total *n* = 361), we observed a consistent empathy and schadenfreude bias driven by team membership. People felt more empathy towards ingroup members than outgroup members and more schadenfreude towards outgroup members. The existence of an intergroup bias did not depend on the nature of the agent: the same effects were observed for human-human and human–robot teams. People reported similar levels of empathy and schadenfreude towards a human and robot player. The human likeness of the robot did not consistently influence this intergroup bias. In other words, similar empathy and schadenfreude biases were observed for both humanoid and mechanoid robots. For all teams, this bias was influenced by the level of team identification; individuals who identified more with their team showed stronger intergroup empathy and schadenfreude bias. Together, we show that similar intergroup dynamics that shape our interactions with people can also shape interactions with robots. Our results highlight the importance of taking intergroup biases into account when examining social dynamics of human-robot interactions.

## Introduction

Robust human-robot relationships require not only robots’ interactive capabilities, but also people’s willingness to accept them as social partners (Dautenhahn, 2007; Hortensius & Cross, 2018). Yet, no matter how human-like robots might become, they are fundamentally machines, which makes them an unprecedented and unique social group in human society. This also brings the possibility that robots will be subjected to social categorisation (Vanman & Kappas, 2019). Researchers in social robotics have emphasised the importance of investigating the impact of social categorisation and intergroup biases on people’s perception and attitudes towards robots (Eyssel & Kuchenbrandt, 2012; Smith et al., in press). Intergroup biases are a prominent consideration in any form of social interaction, since they are linked with two important interpersonal phenomena: empathy and schadenfreude (Cikara et al., 2014; Montalan et al., 2012; Zaki, 2014).

Empathy is a multidimensional construct built from interrelated cognitive and affective components that enables us to perceive and react to the emotional state of others through our own vicarious feelings (Davis, 1983; Eisenberg et al., 1991; Preston & de Waal, 2002). It is known to be a motivational source of engaging in prosocial behaviour but at the same time can also motivate people to hurt others. Empathy is selective and has been associated with a spotlight, where the suffering of one or a few individuals is made more salient than the suffering of the many (Bloom, 2017). The selective nature of empathy can be seen in an intergroup context, where people are found to behave more empathically toward ingroup than outgroup members (Zaki, 2014). This so-called intergroup empathy bias (Cikara et al., 2011) becomes most pronounced in competitive contexts, and is further modulated by the perception that the ingroup and outgroup are separate entities (Cikara et al., 2014) and the likeability of the outgroup members (Cikara and Fiske, 2011). Moreover, empathy is also sensitive to other circumstances, like how much responsibility one bears for one’s pain (Decety et al., 2010).

Another affective response which is influenced by group membership is schadenfreude. While schadenfreude has long been used to describe the malicious pleasure people feel when witnessing the misfortune of others, Feather (1994) was the first to systematically investigate this phenomenon. Several explanations have been offered for why and when schadenfreude exists (Smith et al., 2009). For instance, people tend to feel more schadenfreude towards people who they believe deserve to experience misfortune, possibly by the emotional satisfaction one gets from such deservingness (van Dijk et al., 2005). Furthermore, feelings of envy towards a superior outgroup are also known to enhance feelings of schadenfreude, but only when this envy is malicious in nature (van de Ven et al., 2015). Malicious envy here is characterized by the motivation to pull the other down, while benign envy is characterized by the motivation to improve oneself (van de Ven et al., 2009). Envy also entails feeling inferior to an outgroup, which in turn, also increases feelings of schadenfreude when this outgroup suffers misfortune (Leach & Spears, 2008). A general dislike for a person or a group can be a catalyst for schadenfreude as well (Hareli & Weiner, 2002). Finally, schadenfreude is especially pronounced when the one suffering is an outgroup member (Vanman, 2016) and a stronger ingroup identification further increases feelings of schadenfreude when the outgroup is a rival team (Hoogland et al., 2015).

In the context of human–robot interaction (HRI), studies have shown that people can be induced to include robots in their ingroup by, for instance, giving a robot a name that matched participants’ ethnicity (Eyssel & Kuchenbrandt, 2012), or being assigned to the same team as robots (Fraune et al., 2017). Research from interpersonal settings also provides valuable insights suggesting that categorization of individuals based on arbitrary criteria (e.g., fictive group assignment) can successfully induce intergroup empathy bias (Montalan et al., 2012) and intergroup schadenfreude bias (Cikara et al., 2014). However, the extent to which similar emotional responses caused by intergroup dynamics might also emerge in human–robot teams, and whether people might feel empathy toward an ingroup robot and experience schadenfreude toward an outgroup robot are all open questions. Answers to these questions are important and timely because intergroup biases not only influence the perception of outgroup members, they also restrict interactions with outgroup members (Tajfel & Turner, 1979), thereby reducing the potential of collaboration, education, and care in intergroup settings. Understanding to what extent social intergroup biases positively and negatively shape ongoing interactions with robots is of particular importance to advance collaboration within already existing human-robot teams in the military and in healthcare, for example (Ahn et al., 2015, Broadbent, 2017, Ajoudani et al., 2018, Lakhmani et al., 2020). While potentially these intergroup processes can limit the interaction and collaboration with social robots, they can at the same time serve as a manipulation to increase acceptance of social robots in everyday life and/or in a professional context. For instance, a common ingroup identity can shift perception from an ingroup–outgroup dichotomy to shared group membership (Gaertner et al., 1993). Despite the importance of group interactions in HRI, only a few have ventured into this relatively uncharted area of research.

### Overview of studies

In the present study, we investigated whether similar intergroup empathy and schadenfreude biases exist when humans and robots form a team while competing with a rival human–robot team across a series of one exploratory and three preregistered experiments (see ***Table 1*** for an overview). After a successful validation study to test whether our experimental setup would elicit feelings of empathy and schadenfreude on an interpersonal level (Experiment 1, see the supplementary materials), we continued to verify if, by the current game design, intergroup biases would emerge in human-human teams (Experiment 2) as previous findings indicated (Cikara et al., 2014; Montalan et al., 2012). Finally, Experiments 3 and 4 were designed to probe the effects of intergroup biases in HRI, by adopting two different robot types: one mechanoid or machine-like (Experiment 3: Cozmo robot by Anki, San Fransciso, CA, USA) and one humanoid or more human-like (Experiment 4: Nao robot by SoftBank Robotics, Tokyo, Japan). Executing a series of four experiments allowed us to build evidence in a cumulative manner and to examine the extent to which the effects of intergroup biases generalise across different agents (human players, mechanoid robots, and humanoid robots).

**Table 1:** Study overview of the four experiments.


EXPERIMENT	GOAL	HYPOTHESES		SUPPORTED	PARTICIPANTS	AGENTS	LEVEL

Validation (Experiment 1)	Validate whether people’s feelings are altered based on the result of the game and the focus (self/other) of the question during our competitive game	**1.**	Participants would feel better when they won the game as opposed to when the other player won	Yes	*N* = 81	Human	Interpersonal
		**2.**	Participants would feel worse after they themselves lost as opposed to when the other player lost	Yes			
		**3.**	Participants would feel better when the other player than themselves lost	Yes			

Experiment 2	Verify if intergroup biases would emerge in human-human teams during our competitive game	**1.**	Participants would feel relatively more empathy towards ingroup team members than to outgroup members	Yes	N = 37	Human	Intergroup
		**2.**	Participants would feel more schadenfreude towards the opponents who lost than towards team members who lost.	Yes			

Experiment 3	Investigate whether individuals show intergroup biases towards robots in human-robot teams	**1.**	See experiment 2 – H1	Yes	N = 87	Human & robot (Cozmo)	Intergroup
		**2.**	See experiment 2 – H2	Yes			
		**3.**	More salient intergroup empathy and schadenfreude biases when comparing ingroup and outgroup human players than when comparing the ingroup and outgroup Cozmo robots	No			

Experiment 4	Test if findings generalize across robots who differ in human likeness.	**1.**	See experiment 2 – H1	Yes	N = 93	Human & robot (NAO)	Intergroup
		**2.**	See experiment 2 – H2	Yes			
		**3.**	Increasing tendency to have intergroup biases from the least human-like agent (Cozmo) to a more human-like robot (NAO) and finally the human agent.	No			


Participants in our game scenario were arbitrarily paired with either a robot or a human while playing against a similar team in a competitive reaction time game. A detailed explanation of the aforementioned game scenario can be found in the materials and methods section. This study makes use of a minimal group paradigm (H. Tajfel et al., 1971; Montalan et al., 2012; Otten, 2016), where people form a group based on trivial criteria like aesthetic preferences or by random allocation. We chose this paradigm, as it has already been shown successful in eliciting intergroup empathy and schadenfreude bias, and no existing stereotypes or prejudices towards the ingroup and/or outgroup can influence the results. Previous studies measured participants’ general feelings of empathy and schadenfreude in given scenarios without tracking the dynamics and sources of affective reactions (Cikara et al., 2014; Montalan et al., 2012). Taking into account these factors, we directly assessed people’s trial-by-trial emotional fluctuations by targeting the emotional measures at the level of each player (self, their team member and opponents) for every game outcome (win or lose), while simultaneously considering other influences such as team identification and difference in team scores. To accommodate possible feelings of mixed emotions, we used two independent scales to simultaneously measure participants’ positive affect (feeling good) and negative affect (feeling bad) in response to different players’ game outcomes (adopted from Cikara et al., 2014)— instead of treating positive affect (feeling good) and negative affect (feeling bad) as the two extremes of a continuum. We opted for these scales to assess feelings of empathy and schadenfreude, as they have previously been used to measure the two constructs in online experiments and have proved effective for detecting intergroup biases (Cikara et al., 2014). Furthermore, using the scales in the context in which the ratings were collected, lets us operationalize feelings of empathy (feeling good when someone wins and feeling bad when someone loses) and schadenfreude (feeling good when someone loses).

The overarching hypothesis in the study was that group membership (ingroup/outgroup) impacted people’s empathy and schadenfreude feelings in both human-human (Experiment 2) and human–robot teams (Experiment 3 and 4). Specifically, we expected that people would feel relatively more empathy towards ingroup team members than to outgroup members and would feel more schadenfreude towards the opponents who lost than towards team members who lost. We also hypothesised that an interaction would emerge between group membership (ingroup/outgroup) and human likeness of agents on empathy and schadenfreude. Prior work has suggested that higher human likeness should contribute to larger intergroup biases (Vanman & Kappas, 2019). In accordance, in Experiment 3, we anticipated that intergroup empathy and schadenfreude biases would be more salient in the comparison of ingroup and outgroup human players than in the comparison of the ingroup and outgroup Cozmo robots, whereas in Experiment 4, we expected to see an incremental tendency of intergroup biases caused by the increasing human likeness of the agents (Cozmo < Nao < Human, see the ABOT database *http://www.abotdatabase.info/*, Phillips et al., 2018), with the most extreme intergroup biases found in the most human-like agent (i.e., human) and the least intergroup biases observed in the least human-like agent (i.e., Cozmo).

## Experiment 2

The goal of Experiment 2 was to examine whether an empathy and schadenfreude intergroup bias exists in human teams. If an intergroup empathy bias exists, then we would expect that people would feel more empathy towards their team members (ingroup) than their opponents (outgroup). More precisely, we expected to find a significant interaction between the result of the game (winning or losing the round) and focus (self, team member, opponents) when participants rated how good they felt, whereby participants would report feeling better when ingroup members won a round than when outgroup members won. In addition, we expected to find a similar interaction when participants rated how bad they feel, whereby people would feel worse when ingroup members lost a round than when outgroup members lost. Finally, we hypothesised that people would express more schadenfreude towards outgroup members than ingroup members. This should manifest as participants feeling better when outgroup members lost a round than when ingroup members lost.

## Materials and methods

For Experiment 2, all manipulations, measures and sample size justification and main hypotheses were pre-registered on the Open Science Framework (OSF) before the data was collected (*https://osf.io/2da7u/registrations?view_only=f5625588ab8d4e6693169002d04d447a*).

### Participants

Forty-nine participants (Experiment 2) were recruited via Prolific (*www.prolific.co*). Inclusion criteria for Experiment 2 included country of residency (United Kingdom), previous approval rate on Prolific (100%), and no participation in the previous experiment. After exclusion, the final sample size was 37 participants (27 women, 10 men, 19 to 58 years old, average age 31.92 ± 11.75). Twelve participants were excluded. Nine participants were removed from the analyses because of incomplete data due to technical issues. Following preregistered exclusion criteria, data for three participants were excluded. One of these three participants incurred too many penalties (>15), while the other two did not demonstrate enough variation in responses. The sample size for Experiment 2 was determined after conducting a simulation-based power analysis. Following the guidelines by de Bruine and Barr (2020), we ran simulations based on pilot data (*n* = 9) to make sure that our design had more than .95 power with a .05 alpha error probability to detect a result:focus interaction between ingroup (self+teammate) and outgroup members using linear mixed effects models (Table S20, S21, Figure S13, S14). Participants received £3.20 for their time. We also raffled a £20 bonus payment among the fastest 10% of teams/participants to motivate participants to respond as quickly as possible throughout the game and to create interdependence between the team members in order to increase competition. Participants received written information prior to the study, provided informed consent before the start of the experiment, and were naive to the goal of the study.

### Experimental design

A two (result: win or lose) by three (focus: the participant (self), the participants’ teammate, and both the opponents (outgroup)) factorial within-subjects design was used.

### Competitive reaction time game

**Figure 1:**
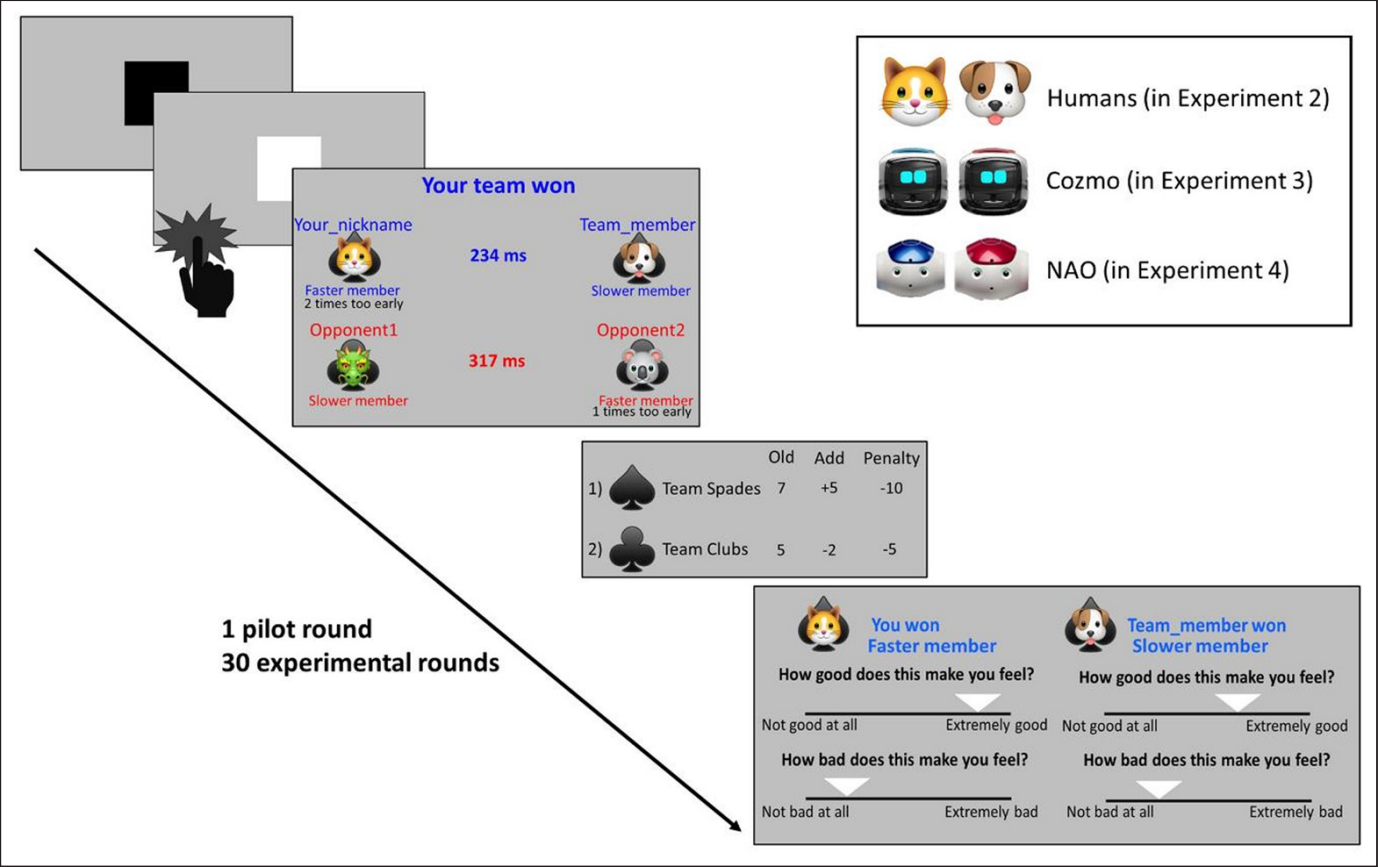
**Competitive reaction time game**. Participants were arbitrarily paired with either a human (Experiment 2), mechanoid Cozmo robot (Experiment 3), or a humanoid Nao robot (Experiment 4) while playing against a similar team in a competitive reaction time game. The game outcome was determined by the average reaction time to the targets per team. The fastest team to respond to the targets won a round and gained five points, while the other team lost two points. We measured participant’s trial-by-trial emotional fluctuations at the level of each player (self, their team member and opponents) for every game outcome (win or lose) while taking into account interpersonal factors (team identification, blame for the result, and score difference). Participant completed two scales that probed trial-by-trial fluctuations in positive affect (feeling good) and negative affect (feeling bad). In Experiment 3 and 4, teams were shuffled every 10 rounds resulting in teams of all possible combinations.

To measure empathy and schadenfreude on a trial-by-trial basis, we devised a competitive reaction time game in which participants competed for points as part of a team (Experiment 2) (***Figure 1***). At the beginning of the experiment, participants chose a nickname and an avatar. This served as a form of identification and gamified the experiment. The participant was then introduced to three players which were also identified by a nickname and an avatar. In reality, all other players except participants themselves were computer programmes, with the nicknames and avatars chosen randomly from a list.

A pilot round served to familiarise the participants with the task and as a way to assign players to one of the two teams. While this assignment was random in nature, participants were told that that they were teamed up with the player whose variation in reaction time (RT) resembled participants’ RT. Participants could then select their team name and corresponding logo (Team Spades/Clubs). This logo was thereafter shown behind each player’s avatar for team identification.

During the competitive reaction time game, participants were asked to respond as fast as possible when the black square in the middle of the screen turned white. After three trials (a round), the team with the lowest average RT won the round and gained five points, while the other team lost two points. We pre-determined that each participant, or their team, would win 60% of the experimental rounds. The faster player/team was shown in blue while the slower player/team was shown in red. Each player was also either labelled as the fastest or the slowest member of the team to manipulate which team member was to blame for losing. The average team RT was calculated based on the RT of the two team members, which was presented on the screen to create participants a feeling of interdependence between the team members. The RT of the participants’ team was made to be either slightly higher or lower than participants’ individual RT to explore the role of perceived responsibility for the game result (e.g., the slower player was blamed more when the team lost). Players would incur a penalty when they responded before the square turned white. This measure was set into place to make people risk-aversive and served as a check to see if people remain attentive during the task. The other players would incur about as many penalties as the participant to make the other players more believable, but also to ensure that the participant would be the winner of the game. After score assignment, participants were able to rate their feelings concerning the outcome of the round.

### Trial-by-trial emotional reactivity

After each round, participants were asked how good and bad the outcome for every player made them feel (on a scales from 0 ‘not good at all’/‘not bad at all’ to 1 ‘extremely good’/‘extremely bad’). Participants responded by moving a slider on the continuous rating scales and were only able to see the labels, but not the values associated to their choice. Feeling good when someone wins and feeling bad when someone loses served as a proxy for empathy, while feeling good when someone loses is seen as a proxy for schadenfreude. At the end of the game, participants were asked how good and bad the final result of the game for every player made them feel.

### Team identification

In Experiment 2, we assessed how much participants identified with their own team and with the opposing team just after team assignment and after the game. We determined this by asking participants three questions per team: “I [value/like/feel] connected to team [Clubs/Spades]” (Cikara et al., 2014). Participants answered these questions using sliders ranging from 0 (‘strongly disagree’) to 1 (‘strongly agree’). Participants responded by moving a slider on the continuous rating scale and were only able to see the labels, but not the values associated with their choice. Higher scores signified greater identification with the teams.

### Procedure

We advertised the experiments as a competitive reaction time game and addressed the study as an investigation on human competitive behaviours. We also provided the fastest 10% of teams/participants with a bonus payment of £20 to incentivise people in the games. After completion 30 rounds in the competitive reaction time game, we asked the participants to describe the other players to check if they believed they were playing games with real humans online. Participants completed the experiment online through Pavlovia (*https://pavlovia.org/*; Peirce et al., 2019) and the experiment took approximately 28 minutes.

### Data processing

All trial-by-trial ratings of emotional reactivity were centered before analyses and were averaged for the opposing team members to create one measure for feeling good or feeling bad for the opposing team (outgroup). The result of the game (result.e: +0.5 = Win, –0.5 = Lose) was effect coded. The focus of the question (focus.f: self, teammate, outgroup) was contrast coded. The first contrast (focus.f1) compared the self with the teammate [self: +1/2, teammate: –1/2, outgroup: 0], while the second contrast (focus.f2) compared the ingroup (self+teammate) with the outgroup [self: +1/3, teammate: +1/3, outgroup: –2/3]. Exploratory analyses compared the outgroup with the self [self: +1/2, teammate: 0, outgroup: –1/2] and the outgroup with the teammate [self: 0, teammate: +1/2, outgroup: –1/2]. For every round, the game result for the self and the teammate was the inverse for the outgroup (as the winning of one team directly translated to the other team losing). To explore the impact of team identification on intergroup biases, we subtracted the average team identification scores of the opposing team from the average scores of the participants’ team, thereby any positive values indicating more identification with their own team and any negative values indicating more identification with the opposing team.

### Analyses

Linear mixed effects models were used to test the possible effects of result (win/lose) and focus (self/teammate/outgroup) on feeling good and feeling bad separately. Analyses were executed with the lme4 package (v1.1.23) in R v4.0.0 (R Core Team, 2020). Post-hoc tests were executed using the emmeans package (v1.4.6). We used an alpha of 0.05 to make inferences concerning our study and controlled for multiple comparisons using Tukey-HSD in post-hoc tests. Model fit was compared using the anova() function.

Based on our validation experiment and pilot data, we preregistered the following model to examine the possible effects of result and focus on feeling good and feeling bad separately: rating ~ result.e * focus.f + (1 + result.e * focus.f | Prolific_ID) + (1 | Ntrail). The participant (Prolific_ID) and trial number (Ntrial) were included as random effects. As specified in the preregistration we checked, besides this model, if a maximal model (with the random effects structure of Ntrial specified as 1 + result.e * focus.f | Ntrial) would converge. As neither of the above models converged, we used an iterative procedure leading to the following model: rating ~ result.e * focus.f + (1 + result.e + focus.f | Prolific_ID) + (1 | Ntrail). To assess if feelings regarding the outcome of a round and the outcome of the entire game are similar to one another, the rating responses at the end of the game were analysed with a linear mixed effects model: rating ~ result.e * focus.e + (1 | Prolific_ID). Slopes are specified maximally (Barr et al., 2013), and participant (Prolific_ID) is included as random effect (Ntrial = 1).

As part of preregistered secondary analyses, we tested the impact of interpersonal factors related to team identification, blame, envy and rivalry. We expected that ingroup identification would be related to stronger feelings of schadenfreude towards outgroup members and empathy towards ingroup members. To test this, we added the difference in team identification to the main models of feeling good and feeling bad. We fitted a less complicated model because of convergence issues: rating ~ difid * result.e * focus.f + (1 + result.e + focus.f | Prolific_ID). Next, we tested if blame, or deservingness to lose, modulated feelings of schadenfreude and empathy. We expected that people would feel more schadenfreude towards the slower outgroup players in losing rounds as they were more to blame, whereas people might feel more empathy towards the slower teammate. To test these predictions, blame, the marking of each player as the faster or slower member of team while taking into account the game result, was added as a fixed effect to the main models of feeling good and feeling bad: rating ~ blame.e * focus.f + (1+focus.f|Prolific_ID) + (1|Ntrial). Finally, we explored if the schadenfreude and empathy biases were modulated by a form of envy and rivalry. The difference in scores served a proxy hereof, as we expected that people would feel more schadenfreude when the other team is ahead of them in points, a source of envy, and people would feel more empathy towards the other team when the participants’ team is far ahead in points, reducing feelings of rivalry. This score difference (difscore.s) was added to a simplified model: rating ~ difscore.s * result.e * focus.f + (1|Prolific_ID) + (1|Ntrial). Models with these interpersonal factors were compared to the main models using the anova() function to test if these factors predicted the trial-by-trial emotional reactivity better.

## Results and Discussion

**Figure 2:**
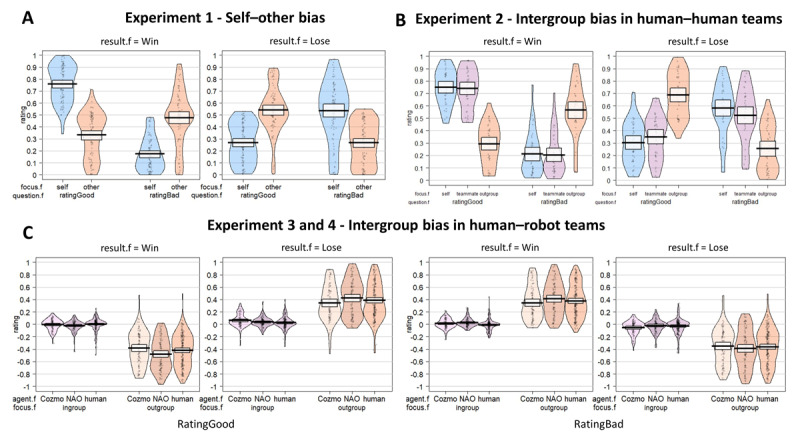
**Trial-by-trial ratings of positive and negative reactions to every game outcome for each player**. A self-other bias was observed when participants played the competitive reaction time game against one player **(A)**. Participants not only felt better when they won and worse when they lost, but also schadenfreude, they felt better when the other player lost a round. A robust empathy and schadenfreude bias driven by team membership was observed (**B** and **C**). Participants felt better when ingroup members won and worse when ingroup members lost (empathy), and felt better when outgroup members lost (schadenfreude). These intergroup schadenfreude and empathy biases were observed when participants formed a team with humans **(B)** and humanoid (NAO) and mechanoid robots (Cozmo) **(C)**. Data is calculated relative to the self for **C**. The dots represent the raw data and the beans the density of the responses. The black bar shows the mean with the white rectangle showing the 95% confidence interval.

For both the trial-by-trial ratings of feeling good and bad, a clear intergroup empathy bias was observed (***Figure 2B*** and Table S3). People felt better when their teammate won (*M* = 0.74, 95% *CI* [0.68–0.79]) than when the outgroup members won (*M* = 0.30, [0.25–0.36], *p* < .001) and felt worse when their teammate lost (*M* = 0.54, [0.47–0.60]) than when outgroup members lost (*M* = 0.26, [0.20–0.33], *p* < .001). In general, participants did not feel better when comparing themselves to their teammate (*β* = 0.02, *p* = .072), but did feel less good towards outgroup members (*β* = –0.05, *p* < .001). Nevertheless, when the participants’ team lost, they felt worse for themselves (*M* = 0.58, [0.51,0.64]) than for their teammate (*p* = .026), whereas they felt less bad for the outgroup members when their team lost (*p* < .0001).

Similarly, people demonstrated an intergroup schadenfreude bias (***Figure 2B*** and Table S3). Participants felt better when outgroup members lost (*M* = 0.68, [0.62–0.74]) as opposed to when the participants themselves (*M* = 0.31, [0.26–0.37], *p* < .001) and their teammate lost (*M* = 0.36, [0.30–0.41], *p* < .001). Yet, they also felt better for their teammate losing compared to themselves losing (*p* = .014). Analysis of the ratings at the end of the game showed the same results (Figure S2 and Table S4). All in all, people tend to feel more empathy towards ingroup members than outgroup members and felt better when misfortune befell outgroup members as opposed to themselves and other ingroup members, while still retaining a self-other bias within their ingroup.

**Figure 3:**
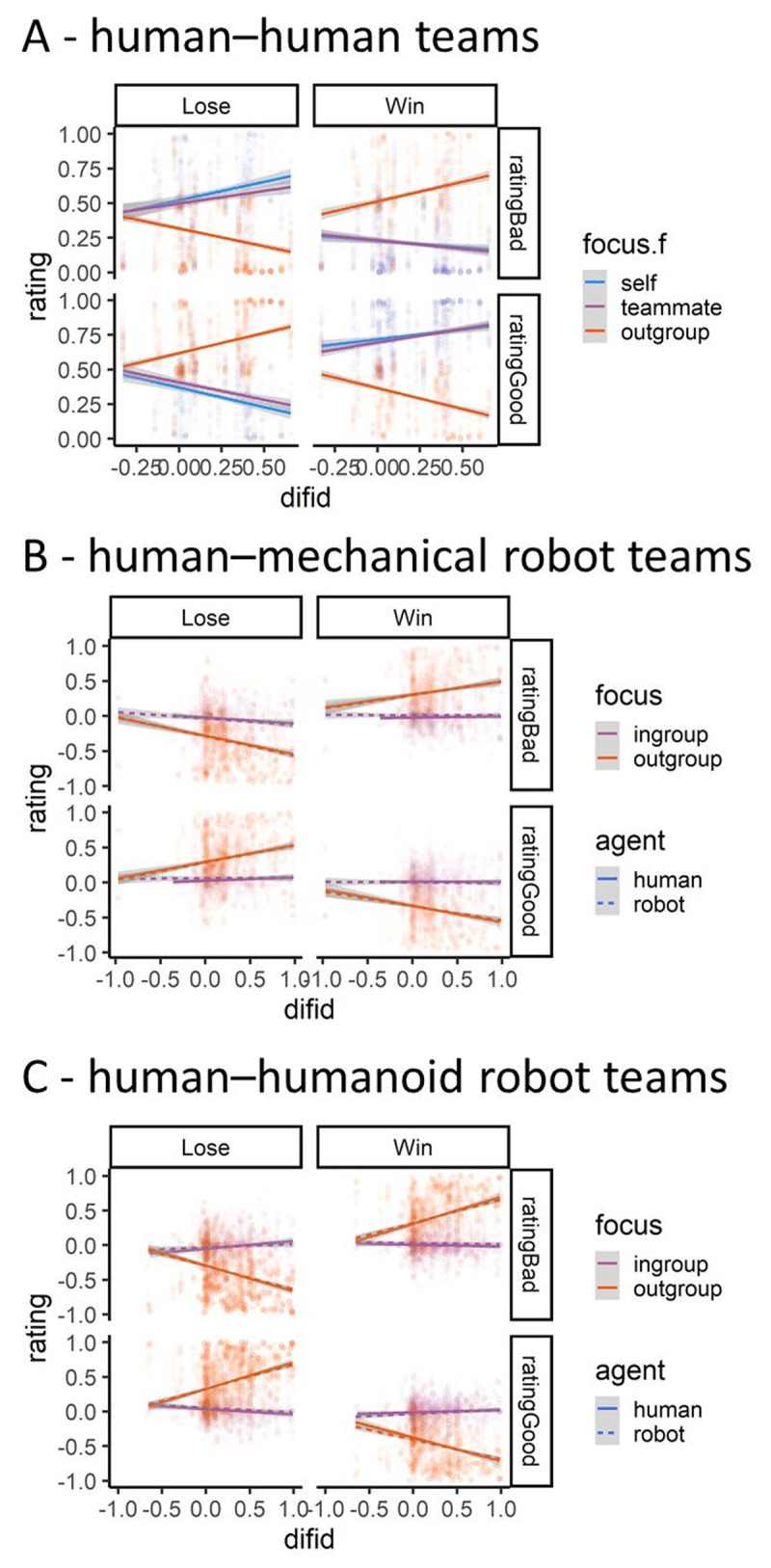
**Team identification consistently increased the intergroup schadenfreude bias**. People who identified more with their team compared to the opponent team (difid) felt more schadenfreude, feeling good when the other team lost. This effect was observed for both human–human **(A)** and human–robot teams **(B–C)**. The points represent the raw data with the linear regression lines of the fitted models with 95% confidence interval around the lines.

As expected, in-group identification increased schadenfreude towards outgroup members and empathy towards ingroup members (***Figure 3A***). Participants who strongly identified with their team not only felt more empathy towards their own team (i.e., feeling worse when they or their teammate lost) but also experienced more schadenfreude to outgroup members (i.e., feeling better when the other team lost). Both the feeling good and bad models improved after adding the difference in team identification (Good: AICmain = –1554, AICteam = –1825, *p* < .001; Bad: AICmain = –1487, AICteam = –1689, *p* < .001), and resulted in a significant interaction between result, ingroup-outgroup contrast and difference in team identification (feeling bad: *β* = 0.87, *p* < .001; feeling good: *β* = 1.03, *p* < .001, Figure S3 and Table S5).

Blame primarily altered how participants rated the feelings for themselves, not for others. Participants felt worse when they were to blame for losing and only felt better for themselves when they were not to blame for losing (Figure S4 and Table S6). However, including the difference in scores did not improve the models for feeling good and bad, which made us suggest that the difference in scores did not consistently influence the empathy and schadenfreude intergroup bias (Figure S5 and Table S7).

## Experiments 3 and 4

The purpose of Experiments 3 and 4 was to investigate whether individuals show intergroup empathy biases towards robots when they team up with one. We also examined the extent to which our findings might generalise across different robots, an unaddressed issue in the field of social robots (Cross et al., 2019; Henschel et al., 2020; Hortensius et al., 2018). In Experiment 3, we used the Cozmo robot, a small entertainment robot with a mechanical design, while we used the NAO robot, a humanoid robot often used in HRI research in Experiment 4. Similar to Experiment 2, we expected to find intergroup biases regardless of agent types – humans or robots. Namely, we predicted there to be a significant interaction between the result of the game (winning or losing) and group membership (ingroup or outgroup). We predicted that participants would express more empathy toward ingroup teammates than toward outgroup members— feeling better when ingroup members win and feeling worse when ingroup members lose; and experience more schadenfreude towards outgroup members than ingroup members — feeling better when outgroup members lose than when ingroup members lose. Based on previous research (Fraune, 2020; Fraune et al., 2017) we also expected the strength of the intergroup biases to depend on the agent types in Experiment 3 and 4. Namely, we expected to find a significant three-way interaction between game results (winning or losing), group membership (ingroup or outgroup) and agent type (human or robot) when participants would rate how good/bad they feel, whereby the intergroup biases would be more pronounced for human players compared to robot players. Finally, as more human-like robots might have the potential to provoke more explicit social categorisation (e.g., Vanman & Kappas, 2019), we anticipated that the levels of human likeness would influence intergroup biases, with higher human likeness leading to bigger intergroup biases (human > Nao > Cozmo). Such an effect should manifest as a significant interaction between game results, group membership and agent type (human, Cozmo, or Nao) when combining data across Experiments 3 and 4.

## Materials and methods

For Experiment 3 and 4, all manipulations, measures and sample size justification and main hypotheses were pre-registered on the Open Science Framework (OSF) before the data was collected: *https://osf.io/nceqp*.

### Participants

One hundred and fourteen (Experiment 3) and one hundred and eleven participants (Experiment 4) were recruited via Prolific (*www.prolific.co*). Inclusion criteria for the country of residency (United Kingdom) to allow for the creation of teams, previous approval rate on Prolific (100%), and no participation in the Experiment 1 or 2 or other ongoing human–robot interaction experiments from the laboratory. In line with preregistered criteria, we excluded the following participants. For Experiment 3, twenty-seven participants were excluded. Fourteen out of them did not complete the experiment, five participants had more than fifteen penalties, eight other participants showed too little variation in response, and another three participants participated twice (we kept data of the completed session). For Experiment 4, eighteen participants were excluded, which included twelve participants who could not finish the experiment, another two who got too many penalties, and the last four who did not use the whole rating scale (the final n for the analyses was 93). Three other participants completed the experiment twice as the experiment crashed during their first try (the data from the completed session were saved and analysed). After exclusion, the final sample size was 87 (45 women, 40 men, 2 unidentified, ranging 18 to 66 years old, average age ± standard deviation: 35.52 ± 12.42 years) for Experiment 3, and 93 (27 women, 33 men, 7 unidentified, 19 to 58 years old, average age 29.11 ± 9.32) for Experiment 4. For both experiments the target sample size was 100 participants. This sample size was informed by the simulation-based power analysis of Experiment 2. Because of the increase in complexity of the design (adding an independent variable of “agent type”), we multiplied the previous sample size of 40 by 2.5. Participants received £3.54 with a £20 bonus payment for the fastest 10% of teams, received written information prior to the study, provided informed consent before start of the experiment, and were naive to the goal of the study.

### Experimental design

A two (results: win or lose) by two (focus or group membership: ingroup or outgroup membership of the other players) by two (agent type: human or robot) factorial within subjects design was used. Similar to Experiment 1 and 2, the analyses were run separately for the feeling factor (good, bad). Additionally, we combined the data from Experiment 3 and 4 to examine if robots’ human-likeness influenced intergroup empathy/schadenfreude biases in human–robot teams (agent type: 3 levels with either a human, Cozmo or Nao player).

### Materials and Procedure

The recruitment procedure was the same as for Experiment 1 and 2. An adapted version of the competitive reaction time game from Experiment 2 was used. Participants were told they would play three games where they teamed up with one of the other players in turns and competing against the other two players. The other three players involved in the game were composed of one human player and two robots (Experiment 3: a red and blue Cozmo, Experiment 4: a red and blue Nao). Before the start of the game participants were introduced to the robots via a short video clip (partly based on the video ‘Meet Cozmo, the AI robot with emotions’ *https://www.youtube.com/watch?v=DHY5kpGTsDE*). The content of the grey-scaled video clip was the same for the two robots/experiments, which involved an illustration of the basic functionality of the robots (e.g. movement and face recognition), a scene showing the robot participating in a similar competitive reaction time game under the pretext that its artificial intelligence engine has been built around all these games to increase the believability of the current game scenario. After the pilot round, participants were told they were sorted into teams based on their response profile. We controlled the order of players who formed teams with participants by these constraints: the first partner was always a robot of either colour (with a 50% chance of each option), the second partner was either a human or another robot (with a 50% chance of each option), and the last partner was the player which had not yet formed a team with the participant. After the pilot round, the participants played three blocks of games, with 10 rounds for each block. The procedures of each block included: 1) team assignment, 2) pre-game team identification measures, 3) ten experimental game rounds with trial-by-trial ratings of emotional reactivity (feeling good/bad), 4) rating of feelings after the final block result, 5) post-game team identification measures. The ratio of winning and losing (60/40) and the distribution of fastest/slowest team member (50/50) remained constant between the three different teams. Similar to the previous experiments, participants could elaborate on who they thought they played against after the final game block. Participants completed the experiment online through Pavlovia (*https://pavlovia.org/*; Peirce et al., 2019) and the experiment took approximately 30 minutes.

### Data processing

For the trial-by-trial emotional reactivity measures, we used the rating towards the self as the baseline of intergroup rating by subtracting this rating from the rating of ingroup and outgroup members. All trial-by-trial ratings were centered before analyses.

The result of the game (result.e: +0.5 = Win, –0.5 = Lose), the focus of the question (group.e: +0.5 = ingroup, –0.5 = outgroup), and type of agent (agent.e: +0.5 = human, –0.5 = agent) were effect coded. When merging the data of Experiment 3 and 4 we used contrast coding for agent type (focus.f = Cozmo, NAO, human). The first contrast (focus.f1) compared Cozmo with NAO [–1/2, +1/2, 0], while the second contrast (focus.f2) compared the NAO with the human [0, –1/2, +1/2]. Within one round of the game, the result of the game for the ingroup is the inverse for the outgroup. The calculation of the indices for team identification were calculated similarly to Experiment 2.

### Analyses

Linear mixed effects models were used to test the predicted interaction between group membership and game result for feeling good and feeling bad separately using a similar approach as in Experiment 1 and 2.

#### Experiment 3

We preregistered the following model with maximally specified slopes (Barr *et al*., 2013) to examine the interaction between result and focus and the interaction between result, focus and agent type on feeling good and feeling bad separately: rating ~ result.e * focus.e * agent.e + (1 + result.e * focus.e * agent | participant_id) + (1 + result.e * focus.e * agent.e | GameRound/trial). The participant (participant_id), game number (GameRound) and trial number (trial) were included as random effects with trial nested in GameRound. The preregistered model did not converge, so less complicated models were fitted to the data. We ended up using the following model for feeling bad: rating ~ result.e * focus.e * agent.e + (1 + result.e * focus.e | participant_id) + (1 | GameRound/trial), and the following model for feeling good: rating ~ result.e * focus.e * agent.e + (1 + result.e * focus.e | participant_id). Another model for feeling good: rating ~ result.e * focus.e * agent.e + (1 + result.e + focus.e | participant_id) + (1 | GameRound/trial), also converged but model comparison showed that the model mentioned first explained more of the data (*p* < .001).

We also looked at the impact of team identification, blame and the difference in team scores on rating in secondary preregistered analyses. Team identification (difid) was added to a less complicated model of feeling bad: rating ~ difid * result.e * focus.e * agent.e + (1 | GameRound/trial), and feeling good: rating ~ difid * result.e * focus.e * agent.e, without participant as random effect because the difference in team identification and participants were closely connected (R2adj = 0.63, *p* < .001). Difference in team scores were added to a slightly less complicated main model of feeling bad: rating ~ difscore.s * result.e * focus.e * agent.e + (1 + result.e * focus.e | participant_id) and feeling good: rating ~ difscore.s * result.e * focus.e * agent.e + (1 + result.e + focus.e | participant_id) + (1 | GameRound/trial). The following model was used to investigate the impact of blame for both feeling good and bad: rating ~ focus.e * agent.e * blameyn.e + (1 + focus.e * blameyn.e | participant_id) + (1 | GameRound/trial).

#### Experiment 4

We preregistered the same model as mentioned in Experiment 3 to examine the interaction between result and focus and the interaction between results, focus and agent type on feeling good and feeling bad in Experiment 4. Similar to Experiment 3, the preregistered model did not converge, so less complicated models were fitted to the data. The following model was used in the analyses for feeling good and bad: rating ~ result.e * focus.e * agent.e + (1 | participant_id) + (0 + result.e * focus.e | participant_id) + (1 | GameRound/trial).

Next, we executed secondary analyses that looked at the influence of team identification, the difference in team scores and blame on rating. Team identification was added to a less complicated model of feeling bad and feeling good: rating ~ difid * result.e * focus.e * agent.e + (1 | GameRound/trial), without participant as a random effect because the difference in team identification and the participant were closely related to one another (R2adj = 0.70, p < .001). Moreover, the difference in team scores were also successfully added to main model of feeling bad: rating ~ difscore.s * result.e * focus.e * agent.e + (1 | participant_id) + (0 + result.e * focus.e | participant_id) + (1 | GameRound/trial), and to a less complicated model of feeling good: rating ~ difid * result.e * focus.e * agent.e + (1 | participant_id) + (0 + result.e * focus.e | participant_id). The impact of blame was assessed by the following model for both feeling good and bad: rating ~ focus.e * agent.e * blameyn.e + (1 + focus.e * blameyn.e | participant_id) + (1 | GameRound/trial).

#### Comparison between Experiments 3 and 4

The last model we preregistered was to examine the interaction between results (winning or losing), group membership (ingroup or outgroup) and agent type (human, Cozmo, or Nao) as a function of human likeness (Cozmo < Nao < human), on how good or bad people felt in the game: rating ~ result.e * focus.f * agent.e + (1 + result.e * focus.f * agent | participant_id) + (1 + result.e * focus.f * agent.e | GameRound/trial). As the preregistered model was unable to converge, we fitted less complicated models to the data for both feeling good and feeling bad: rating ~ result.e * focus.f * agent.e + (1 + result.e * focus.f | participant_id) + (1 | GameRound/trial).

## Results and Discussion

A distinct intergroup empathy bias was found for humans and robots alike for both the trial-by-trial ratings of feeling good and bad in both Experiment 3 and 4 (***Figure 2C, Tables 2*** and ***3***). People felt better when ingroup members won than when outgroup members won (Experiment 3: *p_Human_* < .001; *p_Robot_* < .001; Experiment 4: *p_Human_* < .001; *p_Robot_* < .001) and participants felt relatively worse when ingroup members lost rather than when outgroup members lost (Experiment 3: *p_Human_* < .001; *p_Robot_* < .001; Experiment 4: *p_Human_* < .001; *p_Robot_* < .001). A clear intergroup schadenfreude bias for both humans and robots was observed in both Experiment 3 and 4 (***Figure 2C***). Participants felt better when outgroup members lost as compared to when their ingroup members lost (Experiment 3: *p_Human_* < .001; *p_Robot_* < .001; Experiment 4: *p_Human_* < .001; *p_Robot_* < .001). The occurrence and the strength of the intergroup schadenfreude and empathy bias was independent of agent type (Table S8 and S12). While the strength of the intergroup bias was independent from agent type when feeling bad (Experiment 3: *β* = –0.03, *p* = .123, Experiment 4: *β* = –0.02, *p* = .417), results for feeling good were mixed at first (Experiment 3: *β* = 0.04, *p* = .044, Experiment 4: *β* = 0.01, *p* = .462). Post-hoc tests did, however, not provide a clear difference. In sum, people seem to have a similar intergroup empathy and schadenfreude bias towards humans and robots.

**Table 2:** Trial-by-trial ratings of positive and negative reactions to every game outcome in Experiment 3.


	FEELING GOOD			FEELING BAD	
	
	WIN	LOSE		WIN	LOSE

**Ingroup**					

Human	0.01 [–0.01, 0.03]	0.04 [0.02, 0.06]		–0.02 [–0.04, 0.00]	–0.04 [–0.07, –0.01]

Robot	–0.00 [–0.02, 0.02]	0.06 [0.04, 0.08]		0.01 [–0.00, 0.03]	–0.05 [–0.08, –0.03]

**Outgroup**					

Human	–0.38 [–0.44, –0.33]	0.35 [0.29, 0.41]		0.35 [0.30, 0.40]	–0.34 [–0.40, –0.28]

Robot	–0.38 [–0.44, –0.33]	0.34 [0.28, 0.40]		0.35 [0.29, 0.40]	–0.35 [–0.41, –0.29]


Mean values with 95% confidence intervals are shown.

**Table 3:** Trial-by-trial ratings of positive and negative reactions to every game outcome in Experiment 4.


	FEELING GOOD			FEELING BAD	
	
	WIN	LOSE		WIN	LOSE

**Ingroup**					

Human	–0.00 [–0.02, 0.02]	0.01 [–0.00, 0.03]		–0.00 [–0.02, 0.02]	–0.02 [–0.04, 0.01]

Robot	0.02 [–0.04, –0.00]	0.06 [0.04, 0.08]		0.03 [0.01, 0.04]	–0.02 [–0.04, –0.00]

**Outgroup**					

Human	–0.46 [–0.51, –0.40]	0.42 [0.36, 0.48]		0.40 [0.35, 0.46]	–0.45 [–0.45, –0.32]

Robot	–0.48 [–0.53, –0.43]	0.42 [0.36, 0.48]		0.41 [0.36, 0.47]	–0.39 [–0.45, –0.33]


Mean values with 95% confidence intervals are shown.

Next, we directly compared the intergroup bias found for a mechanoid and humanoid robot. While trial-by-trial ratings of feeling bad did not demonstrate an effect of robot type (*β* = –0.04, *p* =.145), trial-by-trial ratings of feeling good were influenced by robot type (*β* = 0.06, *p* = .025; Table S16). Post-hoc tests of the model suggested that people felt better about ingroup Cozmo losing than ingroup NAO losing.

Similar to Experiment 2, people who identified more with their team felt more schadenfreude towards outgroup members in Experiment 3 and 4 (***Figure 3B*** and ***3C***). An interaction between result, focus, and difference in team identification emerged (Experiment 3: feeling bad: *β* = –0.37, *p* < .001, feeling good: = –0.37, *p* < .001, Experiment 4: feeling bad: *β* = –0.82, *p* < .001, feeling good: *β* = 0.80, *p* < .001; Table S9 and S13, Figure S6 and S9). Team identification for both interactions had a far stronger influence on how participants rated the other team than how they rated their ingroup and this was irrespective of agent type. Those who strongly identified themselves with their team felt better when the other team lost but felt less good when that team won. However, adding the difference in team identification did not consistently improve the fit of the models for the trial-by-trial ratings of feeling good and bad (Experiment 3: AICmain = –2117, AICteam = 3131, *p* < .001; Experiment 4: AICmain = –2717, AICteam = 3192, *p* = 1.000).

Blame did not consistently influence the intergroup empathy and schadenfreude bias across Experiment 3 and 4. While participants felt better about an outgroup human who was blamed for losing (Experiment 3: *M* = 0.37, 95% *CI* [0.31,0.44]) rather than an outgroup human who was not blamed (*M* = 0.32, 95% *CI* [0.26,0.39], *p* = .028) in Experiment 3, this factor of blame was not significant for Experiment 4 (Table S10, S14, Figure S7 and S10). Similarly, including the difference in score did not influence the intergroup empathy and schadenfreude bias (Figure S8, S11, Table S11, S15). Rerunning the analyses while controlling for the belief held by the participants, showed that the intergroup effect holds for both participants who believed they played against a robot and participants who believed they played against a human (Figure S12, Table S17–S19). Interestingly, only people who believed they played against a mechanoid robot showed attenuated schadenfreude and empathy towards this robot, while people who believed they played against a humanoid robot showed increased schadenfreude and empathy towards this robot.

## General Discussion

The goal of the present study was to investigate the presence and dynamics of intergroup biases in human–robot teams. Across one exploratory and three preregistered experiments, we tested the extent to which similar empathy and schadenfreude intergroup biases exist when participants are arbitrarily assigned to human–robot teams as when they are assigned to human-human teams. From these experiments we found robust evidence that team membership influences trial-by-trial emotional reactivity during a competitive reaction time game consistent with an empathy and schadenfreude bias. For both humans and robots alike, people felt more empathy towards ingroup members than outgroup members and felt more schadenfreude towards outgroup members. The level of identification with the team increased these biases consistently across human–robot and human-human teams. Nevertheless, only outgroup humans were subjected to heightened levels of schadenfreude when they could be blamed for encountering misfortune (i.e., when they were the slowest team member of the losing team). Neither the existence nor the strength of an intergroup bias was influenced by whether the agent was a robot or a human. In contrast to expectations, the robot’s human likeness did not influence the strength of the intergroup bias. People did not feel less empathy and increased schadenfreude toward humanoid compared to mechanoid outgroup robots in our study sample. Our results suggest that similar social dynamics and biases determine the subjective feelings towards the agents we collaborate and compete with.

The power of a group membership transcends from human-only teams to teams made up of human and robots. While membership of existing groups (e.g., culture, religion, sports) shapes our social perceptions and behaviour (Stürmer et al., 2005; H. Tajfel & Turner, 1979), assigning people into arbitrary teams can also lead to similar effects (Otten, 2016; H. Tajfel et al., 1971). As indicated by the current findings as well as previous findings (Correia et al., 2018; Eyssel & Kuchenbrandt, 2012; Fraune, 2020; Fraune et al., 2017; Kuchenbrandt et al., 2013), shared team or group membership can foster common group identity that binds humans and robots together which leads to favouring ingroup over outgroup members. This is further proven by the effect of team identification. Consistent with theoretical and empirical accounts (Hoogland et al., 2015; Van Bavel & Cunningham, 2012), increased team identification was related to enhanced intergroup biases in both human-human and human–robot teams. Our results suggest that team membership and identification lead to a general ingroup-favouritism through increased outgroup-dislike, with no clear difference between participants’ emotional reactions to ingroup members and to the self. This is, however, in contrast to previous findings that ingroup human members were favoured over ingroup robot members in terms of negative behavioural outcome (Fraune, 2020; Fraune et al., 2017). As context, behavioural relevance and interdependency are important factors in human–robot interaction (Lyons et al., 2019), a challenge for future research will be to investigate to what extent general ingroup favouritism holds at the perceptual and behavioural level.

The absence of a differential intergroup empathy and schadenfreude bias toward the humanoid and mechanoid robots is contradicting the noted importance of the humanlike appearance of a robot. A wealth of research documents how a robot’s humanlike (or anthropomorphic) appearance can influence the perception, reaction and collaboration with this robot (for a review, see Hortensius & Cross, 2018). Directly comparing the results of a humanoid and mechanoid robot, we found that a robot’s human likeness did not increase the intergroup bias. In contrast to Fraune (2020), we found that people who believed they played against robots showed an even stronger bias towards humanoid robots than humans. As these results are exploratory in nature and the explicit functional role of a robot’s form and shape in the perception and reaction to these agents remain to be understood (Hortensius, Hekele & Cross, 2018), future studies could explore the effect of belief on social cognitive reactions to humanoid and mechanoid robots. Besides the importance of a robot’s appearance, other factors related to social, cognitive, and emotional capabilities of the robot might influence collaboration with a robot. For instance, robots capable of expressing group-based emotions (Correia et al., 2018) and or vulnerability (Traeger et al. 2020) can improve group dynamics.

The similarity in intergroup biases in humans and robots point to the importance of interdependency during human–robot interaction. In contrast to previous studies (Cross et al., 2019; Rosenthal-von der Pütten et al., 2013), we show that people can, in the context of ingroup membership, feel similar levels of empathy towards a robot as towards a fellow human. This could be an important step to advance our understanding of human–robot interaction by comprehensibly modelling social dynamics (Henschel, Hortensius, Cross, 2020), which goes beyond the mere focus of whether robots can be human-like or elicit human-like responses. In the present study, we have done this by including an interdependency between the human user and the robot by performing a task together and competing for resources. With the increased integration of robots into our home and work environments on the near horizon, interdependency between humans and robots remains an important open question. While studies have only just begun to investigate the effect of repeated interactions on social perception and behaviour (Abubshait et al., 2020; Abubshait & Wykowska, 2020; Cross et al., 2019), repeated interactions with a robot are one way to increase interdependency and foster an ingroup mentality. Beyond social interactions, these intergroup dynamics and the power of interdependency are crucial in a professional context as well. Military personnel, first responders, and health care professionals increasingly collaborate with robots as part of their day-to-day work (Broadbent, 2017). It is likely that the relevance and stakes of these human–robot collaborations increase the influence of social dynamics and associated biases.

Productive collaboration requires both parties to shift their attention from individual profit to group gain, and to work collectively as a single social unit (Axelrod, 2006; Pothos et al., 2011). Especially in most situations of real-life collaboration, collaboration entails individuals’ extra effort to maintain cooperative relationships and consistent commitment, as analogised by economic games in social behaviour literature (Pothos et al., 2011; Rapoport & Chammah, 1967). Humans’ willingness to cooperate with robots is consequently pivotal for generating productive human–robot collaborative relationships. Although the differences between humans and robots are fundamental as well as inevitable, our work here demonstrates that it is possible, by giving robots the social category of teammates, for humans to treat robots as social ingroup members, and to experience different emotions due to their robot teammates’ positive or negative situations or outcomes.

One consideration regarding the design of the experiment, is the use of two rating scales to assess empathy and schadenfreude. While these measures have been previously used to assess these two constructs online (Cikara *et al*., 2014), future research could elaborate and verify our findings by using questionnaires which rather make use of direct and indirect statements to gauge empathy and schadenfreude (e.g. van Dijk *et al*., 2005; van de Ven *et al*., 2015). Along the same line, empathy and schadenfreude have both been associated with specific neural responses which could be used to further underline the findings of the current study (Cikara & Fiske, 2011; Molenberghs, 2013).

Another important consideration is the online nature of the present experiments. While online experiments can be robust and lead to reliable results that are comparable to lab-based experiments (Bridges et al., 2020; de Leeuw & Motz, 2016; Miller et al., 2018), and similar biases have been found in screen-based experiments using narratives or ostensible interaction (Cikara et al., 2014), the players in the present experiments were disembodied agents presented on a screen. Embodiment is a crucial factor that drives engagements with other humans (Schilbach et al., 2013) and robots (Wykowska et al., 2016). Physical embodiment of a robot is especially important, as people can have limited understanding of the physical and social capabilities of robots and the initial perception and interaction with these agents can be driven by prior beliefs and expectations (Hortensius & Cross, 2018). Animal avatars were chosen to prevent inherent biases towards gender, age and ethnicity to influence our results, but could in turn have interacted with the cognitive reconstruction of the agents. Usage of human avatars in future research could explore this notion and verify our current findings. The replication across two distinct robots gives us confidence that the finding of ingroup empathy and schadenfreude biases in human–robot teams is not driven by interpersonal variability in perception and cognitive reconstruction of the robot or prior beliefs and expectations thereon. Nevertheless, subtle differences have been found when comparing screen-based to embodied experiments (Willemse & Wykowska, 2019), and we urge future research to replicate these findings with embodied agents in order to truly understand social dynamics during human–robot teams.

## Data Accessibility Statement

Materials, data and code for all experiments are publicly available on the OSF *https://osf.io/ax4dh/*. We report all measures in the study, all manipulations, any data exclusions and the sample size determination rule.

## Additional File

The additional file for this article can be found as follows:

10.5334/joc.177.s1 Supplementary material. Experiments 1 to 4.
