# Supplementary material for: Empathy and Schadenfreude in Human–Robot Teams
Source: J Cogn. 2021 Aug 5;4(1):35. doi: 10.5334/joc.177 (PMC8344963; doi:10.5334/joc.177)
Supplement: Supplementary material. — Experiments 1 to 4. [file joc-4-1-177-s1.pdf]

Supplementary material for

"Empathy and schadenfreude in human–robot teams"

by *Dorina de Jong, Ruud Hortensius, Te-Yi Hsieh & Emily S. Cross*

Experiment 1

Overview of Experiment 1 (pilot)

The purpose of Experiment 1 was to validate whether people's feelings are altered based on the outcome of the game (winning/losing) and the focus (self/other) of the question during a competitive game. As a self-other bias has consistently been reported in the literature (Hoorens, 1995; Humphreys & Sui, 2015; Lindeman, 1997), we expected that participants would feel better when they won the game as opposed to when the other player won, and also feel better when the other player than themselves lost. Moreover, we anticipated that participants would feel worse after they themselves lost as opposed to when the other player lost.

Methods

Participants

Eighty-seven participants were recruited via Prolific (www.prolific.co). Inclusion criteria were nationality (British) and previous approval rate on Prolific (100%). Six participants were excluded. Three participants did not complete the questionnaire regarding dispositional empathy, one participant was not able to see the rating screens in the last 12 rounds, one other participant had more than fifteen penalties and another participant participated twice (we kept data from the first occurrence). After exclusion, the final sample size was 81 participants (48 women, 33 men, 18 to 63 years old, average age ± standard deviation: 32.02±11.03 years). Participants received £2.10 for their time. We also raffled a £20 bonus payment among the fastest 10% of teams/participants to motivate participants to respond as quickly as possible throughout the game. Participants received written information prior to the study, provided informed consent before the start of the experiment, and were naive to the goal of the study.

Experimental design

A two (result: win or lose) by two (focus: the participant (self), and the opponent) factorial within-subjects design was used.

Competitive reaction time game

Participants of Experiment 1 did a highly similar competitive reaction time game with regards to the other experiments. Nevertheless, there was only one opponent, and the participants were not put in a team together with another player.

Procedure

Participants completed 25 rounds in the competitive reaction time game, before describing the other player to check if they believed they were playing games with real humans online. Participants also completed the interpersonal reactivity index (IRI; Davis, 1980, 1983) to measure dispositional empathy. This questionnaire includes four subscales measuring perspective-taking (the cognitive ability to take someone's viewpoint or perspective), fantasy (the ability to identify with a fictitious character), empathic concern (other-oriented feelings of sympathy and concern in response to someone else suffering) and personal distress (self-oriented feelings of distress and unease in response to someone else suffering). Participants completed the experiment online through Pavlovia (https://pavlovia.org/; Peirce et al., 2019) , as well as formr (IRI questionnaire in Experiment 1, https://formr.org/; Arslan et al., 2020) and the experiment took approximately 28 minutes.

Data processing

The result of the game (result.e: +0.5 = Win, -0.5 = Lose) and the focus of the question (focus.e: +0.5 = self, -0.5 = other) were both effect coded.

Analyses

We were especially interested in exploring the interaction between result and focus on rating, and therefore ran the following mixed effects model: rating ~ result.e*focus.e + (1+result.e *focus.e|Prolific_ID) + (1|Ntrial) – on participants' feeling good and bad rating. The participant (Prolific_ID) and trial number (Ntrial) were included as random effects and the random slopes were simplified until the model converged. To examine the influence of dispositional empathy on trial-by-trial emotional reactivity, we used linear multiple regression models for feeling good and bad separately with the IRI subscales as predictors.

Results and discussion

People felt better when they won (M = 0.76, 95% CI [0.73-0.79]) and felt worse when they lost (M = 0.54, 95% CI [0.48,0.59]) in comparison to the other player (Mwon = 0.32, 95% CI [0.28-0.37], Mlose = 0.27, 95% CI [0.22,0.30], Figure 2A). The results of a linear mixed-effects model for feeling bad showed that result (bad: β = -0.08, $p < .001$, good: β = 0.14, $p < .001$), focus (bad: β = -0.02, p = .027, good: β = 0.08, $p < .001$), and the interaction between result and focus (bad: β = -0.59, $p < .001$, good: β =0.72, $p < .001$) all had an impact on how good or bad people felt about the outcome of the round (Table S1). More importantly, the experimental setup caused people to feel schadenfreude towards the other player. People felt better when the other player lost a round (M = 0.48, 95% CI [0.44-0.53]) as opposed to when they lost (M = 0.17, 95% CI [0.14-0.20], $p < .001$), thus people tended to feel relatively better when misfortune befalls someone else. Results of a multiple regression showed that dispositional empathy did not modulate trial-by-trial emotional reactivity ($F(4,619) = 1.16$, p = .328, $R^2_{adj} = 0.001$). None of the IRI subscales had a significant impact on how participants rated the feelings of themselves and others (Figure S1 and Table S2).

Main analyses

rating ~ result*focus + (1+result*focus|subject) + (1|Ntrial)

	rating.c				rating.c			
Predictors	*Estimates*	*CI*	*Statistic*	*p*	*Estimates*	*CI*	*Statistic*	*p*
(Intercept)	-0.06	-0.09 – -0.03	-3.55	**<0.001**	0.05	0.03 – 0.07	4.66	**<0.001**
result.e	-0.08	-0.10 – -0.06	-8.20	**<0.001**	0.14	0.12 – 0.17	10.81	**<0.001**
focus.e	-0.02	-0.03 – -0.00	-2.22	**0.027**	0.08	0.06 – 0.10	6.90	**<0.001**
result.e * focus.e	-0.59	-0.70 – -0.48	-10.23	**<0.001**	0.72	0.61 – 0.83	12.61	**<0.001**
**Random Effects**								
σ^2	0.03				0.02			
τ_{00}	0.02 Prolific_ID				0.01 Prolific_ID			
	0.00 Ntrial.f				0.00 Ntrial			
τ_{11}	0.01 Prolific_ID.result.e				0.01 Prolific_ID.result.e			
	0.00 Prolific_ID.focus.e				0.01 Prolific_ID.focus.e			
	0.26 Prolific_ID.result.e:focus.e				0.26 Prolific_ID.result.e:focus.e			
ρ_{01}	-0.33 Prolific_ID.result.e				-0.30 Prolific_ID.result.e			
	-0.14 Prolific_ID.focus.e				-0.49 Prolific_ID.focus.e			
	-0.47 Prolific_ID.result.e:focus.e				-0.14 Prolific_ID.result.e:focus.e			
ICC	0.57				0.58			
N	81 Prolific_ID				81 Prolific_ID			
	25 Ntrial.f				25 Ntrial			
Observations	4050				4050			
Marginal R^2 / Conditional R^2	0.266 / 0.684				0.436 / 0.763			

Table S1. Model summaries of LMEMs of ratingBad and ratingGood for Experiment 1. Estimated model of ratingBad (left) and rating good (right) with fixed and random effects with 95% confidence intervals (CI). The fixed effects are result (win, lose) and focus (self, other). Intraclass correlation (ICC) depicts how much of the total variance is accounted for by clustering. The marginal R-squared value only considers the variance of the fixed effects, while the conditional R-squared takes both the fixed and random effects in account. Fitted model is shown above the table. Fitted model is shown above the table and is the same for ratingBad as ratingGood.

Exploratory analyses – Dispositional empathy

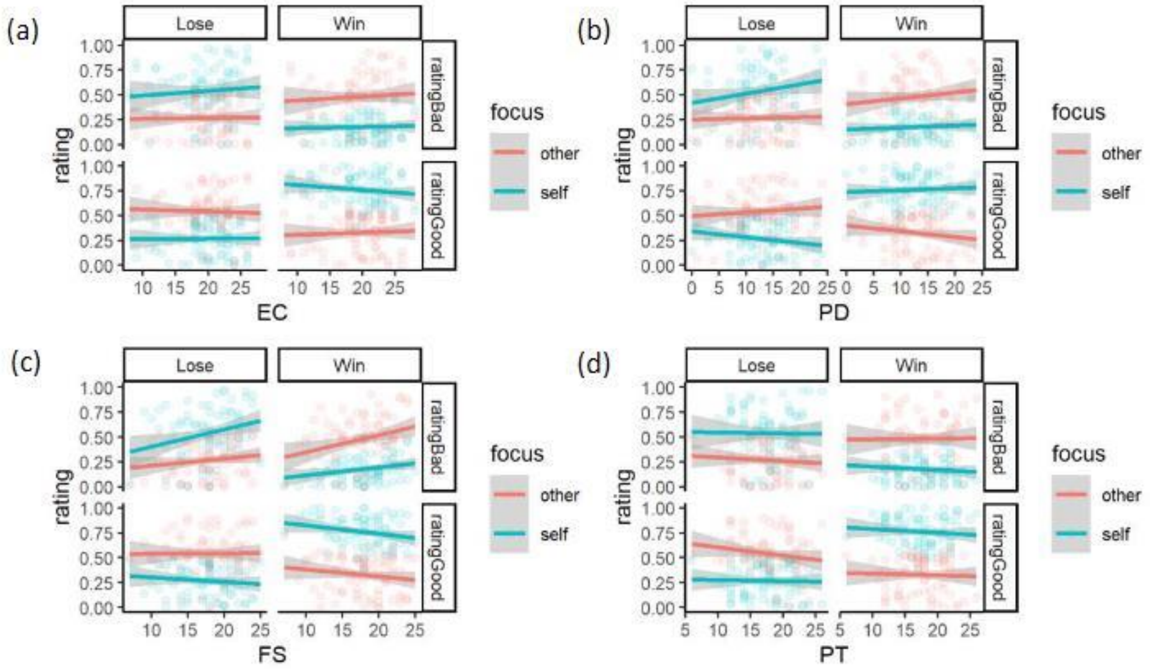

Figure S1. Relation between feeling good and bad and the IRI-subscales. (a) Relation between feeling good and feeling bad and empathic concern. (b) Relation between feeling good and feeling bad and personal distress. (c) Relation between feeling good and feeling bad and fantasy scale. (d) Relation between feeling good and feeling bad and perspective taking.

Rating.s ~ EC + PD + PT + FS

	rating			
Predictors	*Estimates*	*CI*	*Statistic*	*p*
(Intercept)	0.40	0.28 – 0.52	6.55	**<0.001**
EC	-0.00	-0.01 – 0.01	-0.02	0.984
PD	0.00	-0.00 – 0.00	0.29	0.772
PT	-0.00	-0.01 – 0.00	-1.44	0.150
FS	0.01	-0.00 – 0.01	1.53	0.127
Observations	624			
R^2 / R^2 adjusted	0.007 / 0.001			

Table S2. Model summary of multiple regression model for dispositional empathy. Estimated model of rating with fixed effects of measures of dispositional empathy with 95% confidence intervals (CI). Measures of dispositional empathy are empathic concern (EC), personal distress (PD), perspective taking (PT) and fantasy scale (FS). None of the subscales shared multicollinearity (EC: VIF=1.72; PD: VIF =1.12; PT: VIF=1.32; FS: VIF=1.53), which means that none of the independent variables in the model are correlated with one another and do not explain the same variance in the model.

Experiment 2

Main analyses

rating ~ result*focus + (1+result+focus|subject) + (1|Ntrial)

	rating.s				rating.s			
Predictors	*Estimates*	*CI*	*Statistic*	*p*	*Estimates*	*CI*	*Statistic*	*p*
(Intercept)	-0.06	-0.10 – -0.01	-2.64	**0.008**	0.07	0.05 – 0.10	5.49	**<0.001**
result.e	-0.13	-0.16 – -0.10	-8.01	**<0.001**	0.15	0.11 – 0.18	8.63	**<0.001**
focus.f1	-0.02	-0.04 – -0.00	-2.52	**0.012**	0.02	-0.00 – 0.03	1.80	0.072
focus.f2	0.04	0.01 – 0.06	2.69	**0.007**	-0.05	-0.06 – -0.03	-5.13	**<0.001**
result.e : focus.f1	0.04	0.01 – 0.07	2.29	**0.022**	-0.06	-0.09 – -0.02	-3.28	**0.001**
result.e : focus.f2	0.66	0.63 – 0.68	54.09	**<0.001**	-0.78	-0.81 – -0.76	-64.29	**<0.001**
**Random Effects**								
σ^2	0.04				0.04			
τ_{00}	0.01 Prolific_ID				0.00 Prolific_ID			
	0.00 Ntrial				0.00 Ntrial			
τ_{11}	0.01 Prolific_ID.result.e				0.01 Prolific_ID.result.e			
	0.00 Prolific_ID.focus.f1				0.00 Prolific_ID.focus.f1			
	0.01 Prolific_ID.focus.f2				0.00 Prolific_ID.focus.f2			
ρ_{01}	-0.08 Prolific_ID.result.e				-0.47 Prolific_ID.result.e			
	-0.36 Prolific_ID.focus.f1				0.08 Prolific_ID.focus.f1			
	-0.18 Prolific_ID.focus.f2				-0.54 Prolific_ID.focus.f2			
ICC	0.33				0.17			
N	37 Prolific_ID				37 Prolific_ID			
	30 Ntrial				30 Ntrial			
Observations	4440				4440			
Marginal R^2 / Conditional R^2	0.316 / 0.540				0.445 / 0.542			

Table S3. Model summaries of LMEMs of ratingBad and ratingGood for Experiment 2. (a) Estimated model of ratingBad (left) and rating good (right) fixed and random effects with 95% confidence intervals (CI) of experiment 2. The fixed effects are result (win, lose) and focus (self, teammate, outgroup). Focus.f1 compared the self with the teammate, while the second contrast (focus.f2) compared the ingroup (self+teammate) with the outgroup. Intraclass correlation (ICC) depicts how much of the total variance is accounted for by clustering. The marginal R-squared value only considers the variance of the fixed effects, while the conditional R-squared takes both the fixed and random effects in account. Fitted model of both ratingGood and ratingBad is shown above the table.

Exploratory analyses – Rating at the end of the game

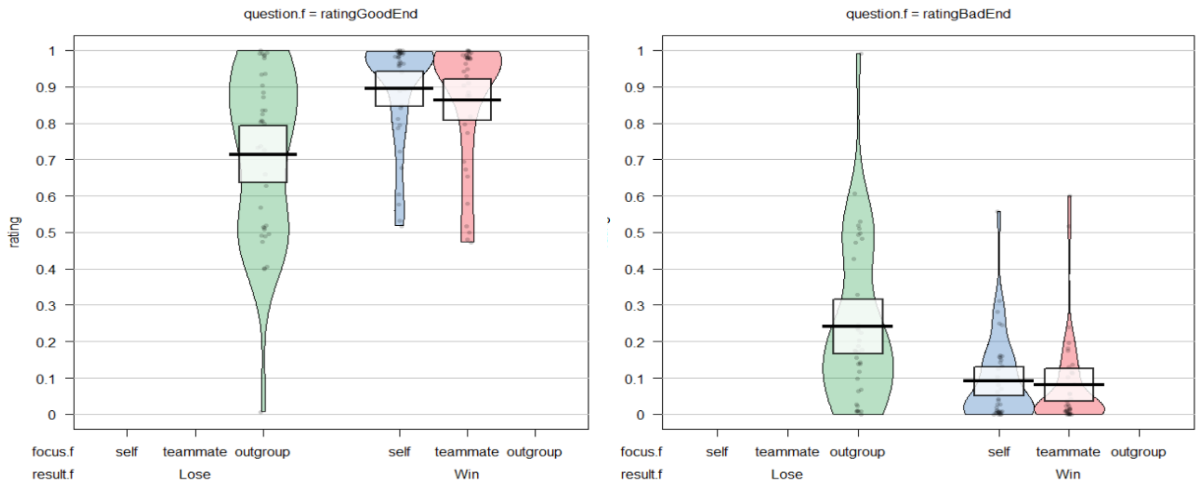

Figure S2. Visualisation of rating for feeling bad and feeling good at the end of Experiment 2. Rating responses for feeling good (left) and for feeling bad (right) for every outcome and player at the end of the game. The dots represent the raw data and the beans the density of the responses. The black bar shows the mean with the white rectangle showing the 95% confidence interval. All participants won their game, so the other team always lost.

*rating.s ~ result.e * focus.f + (1+focus.f|Prolific_ID)*

	rating.s				rating.s			
Predictors	*Estimates*	*CI*	*Statistic*	*p*	*Estimates*	*CI*	*Statistic*	*p*
(Intercept)	-0.29	-0.33 – -0.24	-12.27	**<0.001**	0.35	0.30 – 0.40	13.26	**<0.001**
result.e	-0.15	-0.23 – -0.08	-4.30	**<0.001**	0.16	0.09 – 0.24	4.29	**<0.001**
focus.f1	-0.01	-0.04 – 0.01	-0.82	0.411	-0.03	-0.06 – 0.00	-1.95	0.051
**Random Effects**								
σ^2	0.00				0.00			
τ_{00}	0.02 Prolific_ID				0.02 Prolific_ID			
τ_{11}	0.01 Prolific_ID.focus.f1				0.01 Prolific_ID.focus.f1			
	0.05 Prolific_ID.focus.f2				0.05 Prolific_ID.focus.f2			
ρ_{01}	0.17				0.24			
	0.36				0.20			
ICC	0.98				0.99			
N	37 Prolific_ID				37 Prolific_ID			
Observations	1520				1520			
Marginal R^2 / Conditional R^2	0.265 / 0.985				0.231 / 0.989			

Table S4. Model summaries of LMEMs of ratingBad and ratingGood at the end of experiment 2. (a) Estimated model of ratingBad (a) and rating good (b) fixed and random effects with 95% confidence intervals (CI) at the end of experiment 2. The model dropped three columns as the fixed-effect model matrix is rank deficient. The remaining fixed effects were result (win, lose) and focus.f1 which compared the self with the teammate. Intraclass correlation (ICC) depicts how much of the total variance is accounted for by clustering. The marginal R-squared value only considers the variance of the fixed effects, while the conditional R-squared takes both the fixed and random effects in account. Fitted model of both ratingGood and ratingBad is shown above the table.

Exploratory analyses – Team identification

We checked whether assigning people to arbitrary teams led them to identify more with their team than the opposing team. Indeed, participants identified themselves more with their own team (before: 0.70 [0.65-0.75], after: 0.77 [0.70-0.83]) than the other team (before: 0.48 [0.41-0.54], after: 0.49 [0.42-0.55], β = -0.22, p<.001). Team identification did not change over time (β = 0.07, p=0.114) and there was also no interaction between time and group (β = -0.06, p=0.329; see Figure). This implies that arbitrarily assigning people to teams causes people to readily identify with their team, and this remains stable over time.

(a)

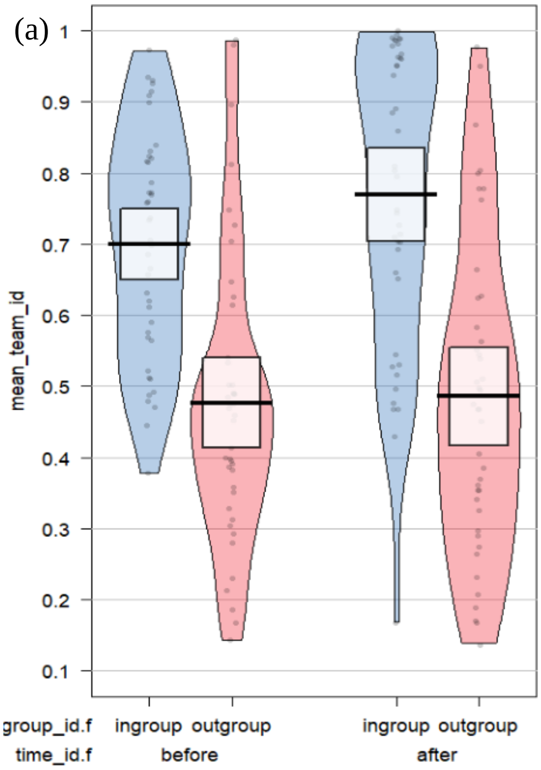

(b)

*Mean_team_id ~ time_id * group_id*

	**mean_team_id**		
Predictors	*Estimates*	*CI*	*p*
(Intercept)	0.70	0.64 – 0.76	**<0.001**
time_id.f [after]	0.07	-0.02 – 0.16	0.114
group_id.f [outgroup]	-0.22	-0.31 – -0.14	**<0.001**
time_id.f [after] * group_id.f [outgroup]	-0.06	-0.18 – 0.06	0.329
Observations	168		
R^2 / R^2 adjusted	0.298 / 0.286		

Figure S3. Team identification before and after the game in Experiment 2. (a) Plot of team identification for the own team (blue) and opposing team (red) before and after the game in Experiment 2. The dots represent the raw data and the beans the density of the responses. The black bar shows the mean with the white rectangle showing the 95% confidence interval. (b) Estimated model summary of multiple regression model with a fixed effect of time_id (before or after the game) and group_id (ingroup or outgroup team) with 95% confidence intervals (CI).

*rating ~ difid * result.e * focus.f + (1 + result.e + focus.f | Prolific_ID)*

	**rating.s**				**rating.s**			
Predictors	*Estimates*	*CI*	*Statistic*	*p*	*Estimates*	*CI*	*Statistic*	*p*
(Intercept)	-0.06	-0.12 – -0.01	-2.33	**0.020**	0.08	0.05 – 0.11	5.20	**<0.001**
difid	0.04	-0.13 – 0.22	0.47	0.637	-0.03	-0.13 – 0.06	-0.67	0.505
result.e	-0.12	-0.16 – -0.08	-5.62	**<0.001**	0.13	0.08 – 0.17	5.56	**<0.001**
focus.f1	-0.01	-0.03 – 0.01	-0.94	0.349	0.01	-0.02 – 0.03	0.64	0.525
focus.f2	0.05	0.01 – 0.08	2.47	**0.013**	-0.06	-0.08 – -0.03	-4.55	**<0.001**
difid * result.e	-0.04	-0.18 – 0.10	-0.60	0.547	0.09	-0.05 – 0.24	1.27	0.205
difid : focus.f1	-0.05	-0.13 – 0.02	-1.45	0.146	0.04	-0.04 – 0.12	1.06	0.291
difid : focus.f2	-0.04	-0.16 – 0.07	-0.73	0.466	0.04	-0.03 – 0.12	1.09	0.276
result.e : focus.f1	0.03	-0.02 – 0.07	1.19	0.233	-0.06	-0.11 – -0.02	-2.73	**0.006**
result.e : focus.f2	0.48	0.44 – 0.51	29.83	**<0.001**	-0.57	-0.60 – -0.54	-35.97	**<0.001**
difid : result.e : focus.f1	0.06	-0.09 – 0.20	0.80	0.423	0.03	-0.12 – 0.17	0.34	0.731
difid : result.e : focus.f2	0.87	0.77 – 0.97	16.82	**<0.001**	-1.03	-1.13 – -0.93	-20.02	**<0.001**
**Random Effects**								
σ^2	0.04				0.04			
τ_{00}	0.01 $_{Prolific\_ID}$				0.00 $_{Prolific\_ID}$			
τ_{11}	0.01 $_{Prolific\_ID.result.e}$				0.01 $_{Prolific\_ID.result.e}$			
	0.00 $_{Prolific\_ID.focus.f1}$				0.00 $_{Prolific\_ID.focus.f1}$			
	0.01 $_{Prolific\_ID.focus.f2}$				0.00 $_{Prolific\_ID.focus.f2}$			
ρ_{01}	-0.08				-0.46			
	-0.52				0.04			
	-0.18				-0.56			
ICC	0.33				0.16			
N	37 $_{Prolific\_ID}$				37 $_{Prolific\_ID}$			
Observations	4440				4440			
Marginal R^2 / Conditional R^2	0.342 / 0.559				0.484 / 0.565			

Table S5: Model summaries of LMEMs of ratingBad and ratingGood when including the difference in team identification in Experiment 2. (a) Estimated model of ratingBad (left) and rating good (right) fixed and random effects with 95% confidence intervals (CI) of experiment 2 while including the difference in team identification (difid). The other fixed effects included result (win, lose) and focus (self, teammate, outgroup). Focus.f1 compared the self with the teammate, while the second contrast (focus.f2) compared the ingroup (self+teammate) with the outgroup. Intraclass correlation (ICC) depicts how much of the total variance is accounted for by clustering. The marginal R-squared value only considers the variance of the fixed effects, while the conditional R-squared takes both the fixed and random effects in account. Fitted model of both ratingGood and ratingBad is shown above the table.

Exploratory analyses – Blame

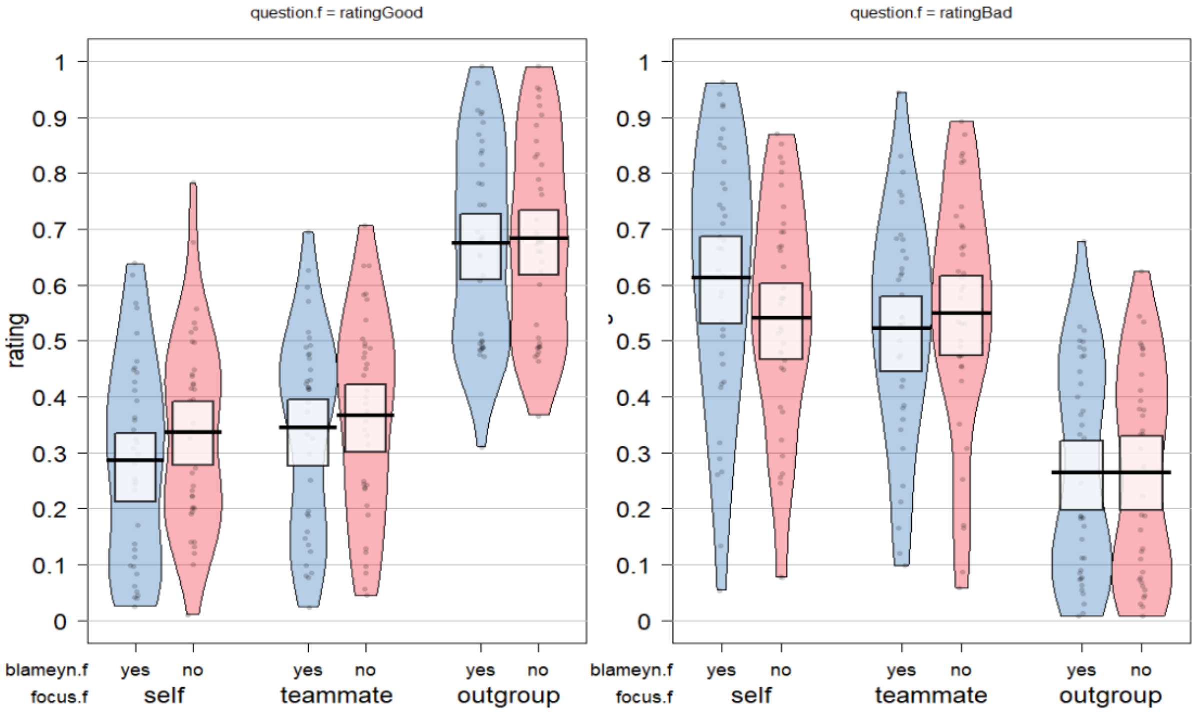

Figure S4. Visualization of relation between blame and rating in Experiment 2. Plot of the effect of blame, with yes (this person is the one to blame for losing) in blue and no (this person is not the one to blame for losing) in red, on ratingGood (left) and ratingBad (right) when losing. The dots represent the raw data and the beans the density of the responses. The black bar shows the mean with the white rectangle showing the 95% confidence interval.

*rating ~ blameyn.e * focus.f + (1+focus.f|Prolific_ID) + (1|Ntrial)*

	rating.s				rating.s			
Predictors	*Estimates*	*CI*	*Statistic*	*p*	*Estimates*	*CI*	*Statistic*	*p*
(Intercept)	0.01	-0.04 – 0.06	0.40	0.689	-0.00	-0.03 – 0.03	-0.05	0.963
focus.f1	-0.04	-0.07 – -0.01	-3.02	**0.003**	0.04	0.02 – 0.07	3.35	**0.001**
focus.f2	-0.29	-0.38 – -0.20	-6.35	**<0.001**	0.35	0.26 – 0.44	7.52	**<0.001**
blameyn.e	0.02	0.00 – 0.03	2.15	**0.031**	-0.03	-0.04 – -0.01	-3.74	**<0.001**
focus.f1 * blameyn.e	-0.09	-0.13 – -0.05	-4.67	**<0.001**	0.02	-0.02 – 0.06	1.08	0.282
focus.f2 * blameyn.e	-0.02	-0.05 – 0.00	-1.74	0.081	0.03	0.00 – 0.05	2.28	**0.023**
**Random Effects**								
σ^2	0.02				0.02			
τ_{00}	0.02 $_{Prolific\_ID}$				0.01 $_{Prolific\_ID}$			
	0.00 $_{Ntrial}$				0.00 $_{Ntrial}$			
τ_{11}	0.00 $_{Prolific\_ID.focus.f1}$				0.00 $_{Prolific\_ID.focus.f1}$			
	0.08 $_{Prolific\_ID.focus.f2}$				0.08 $_{Prolific\_ID.focus.f2}$			
ρ_{01}	0.02 $_{Prolific\_ID.focus.f1}$				0.15 $_{Prolific\_ID.focus.f1}$			
	-0.39 $_{Prolific\_ID.focus.f2}$				-0.39 $_{Prolific\_ID.focus.f2}$			
ICC	0.62				0.57			
N	37 $_{Prolific\_ID}$				37 $_{Prolific\_ID}$			
	30 $_{Ntrial}$				30 $_{Ntrial}$			
Observations	2220				2220			
Marginal R^2 / Conditional R^2	0.259 / 0.720				0.367 / 0.729			

Table S6: Model summaries of LMEMs of ratingBad and ratingGood when including blame in Experiment 2. Estimated model of feeling bad (left) and feeling good (right) fixed and random effects with 95% confidence intervals (CI) of experiment 2 while including blame (blameyn.e). The other fixed effects included result (win, lose) and focus (self, teammate, outgroup). Focus.f1 compared the self with the teammate, while the second contrast (focus.f2) compared the ingroup (self+teammate) with the outgroup. Intraclass correlation (ICC) depicts how much of the total variance is accounted for by clustering. The marginal R-squared value only considers the variance of the fixed effects, while the conditional R-squared takes both the fixed and random effects in account. Fitted model of both ratingGood and ratingBad is shown above the table.

Exploratory analyses – Difference scores

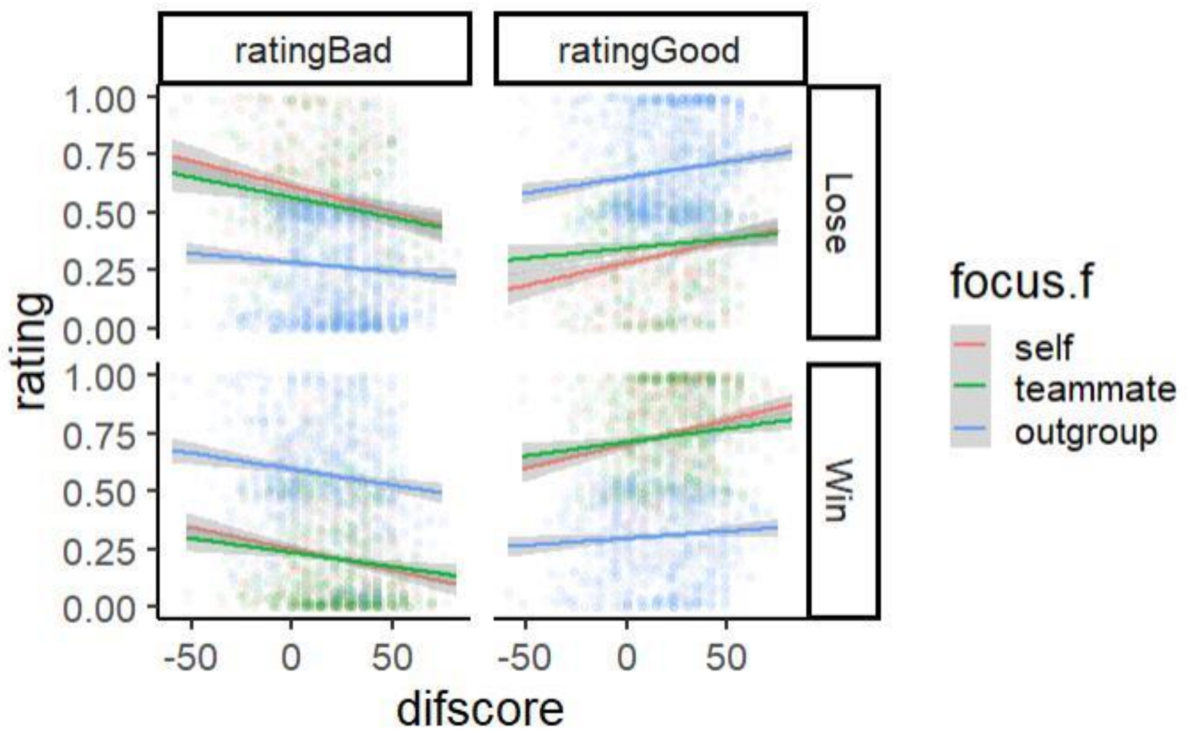

Figure S5. Visualization of relation between rating and the difference in scores in Experiment 2. Plot shows the raw data and linear regression lines of the fitted models with 95% confidence intervals. This is shown for all given conditions: result (Win/Lose), focus (self/teammate/outgroup) and the valence of the question (ratingBad/ ratingGood).

*rating ~ difscore.s * result.e * focus.f + (1|Prolific_ID) + (1|Ntrial)*								
	**rating.s**				**rating.s**			
Predictors	*Estimates*	*CI*	*Statistic*	*p*	*Estimates*	*CI*	*Statistic*	*p*
(Intercept)	-0.03	-0.07 – 0.01	-1.29	0.197	0.04	0.02 – 0.07	3.40	**0.001**
difscore.s	-0.16	-0.21 – -0.11	-6.63	**<0.001**	0.16	0.11 – 0.20	6.64	**<0.001**
result.e	-0.13	-0.14 – -0.11	-14.75	**<0.001**	0.14	0.13 – 0.16	16.95	**<0.001**
focus.f1	-0.03	-0.05 – -0.01	-2.75	**0.006**	0.03	0.01 – 0.06	3.05	**0.002**
focus.f2	0.02	0.01 – 0.04	2.90	**0.004**	-0.04	-0.05 – -0.02	-4.60	**<0.001**
difscore.s * result.e	0.01	-0.04 – 0.07	0.44	0.656	-0.01	-0.07 – 0.04	-0.49	0.627
difscore.s : focus.f1	0.05	-0.02 – 0.13	1.34	0.179	-0.10	-0.18 – -0.02	-2.57	**0.010**
difscore.s : focus.f2	0.07	0.01 – 0.12	2.40	**0.016**	-0.05	-0.11 – 0.00	-1.92	0.055
result.e : focus.f1	0.03	-0.01 – 0.08	1.39	0.164	-0.05	-0.10 – -0.01	-2.33	**0.020**
result.e : focus.f2	0.66	0.63 – 0.69	39.97	**<0.001**	-0.75	-0.78 – -0.72	-45.85	**<0.001**
difscore.s : result.e : focus.f1	0.02	-0.14 – 0.17	0.24	0.812	0.02	-0.13 – 0.17	0.24	0.808
difscore.s : result.e : focus.f2	-0.13	-0.24 – -0.02	-2.23	**0.026**	-0.07	-0.17 – 0.04	-1.17	0.242
**Random Effects**								
σ^2	0.04				0.04			
τ_{00}	0.01 Prolific_ID				0.00 Prolific_ID			
	0.00 Ntrial				0.00 Ntrial			
ICC	0.25				0.10			
N	37 Prolific_ID				37 Prolific_ID			
	30 Ntrial				30 Ntrial			
Observations	4440				4440			
Marginal R^2 / Conditional R^2	0.328 / 0.499				0.456 / 0.513			

Table S7: Model summaries of LMEMs of ratingBad and ratingGood when including the difference in scores in Experiment 2. (a) Estimated model of ratingBad (left) and rating good (right) fixed and random effects with 95% confidence intervals (CI) of experiment 2 while including the difference in scores as fixed-effect. The other fixed effects were result (win, lose) and focus (self, teammate, outgroup).Focus.f1 compared the self with the teammate, while the second contrast (focus.f2) compared the ingroup (self+teammate) with the outgroup. Intraclass correlation (ICC) depicts how much of the total variance is accounted for by clustering. The marginal R-squared value only considers the variance of the fixed effects, while the conditional R-squared takes both the fixed and random effects in account. Fitted model of both ratingGood and ratingBad is shown above the table.

Experiment 3

Main analyses

*Bad: rating ~ result.e * focus.e * agent.e + (1 + result.e * focus.e | participant_id) + (1 | GameRound/trial)*
*Good: rating ~ result.e * focus.e * agent.e + (1 + result.e * focus.e | participant_id)*

	**ratingsc**				**ratingsc**			
Predictors	*Estimates*	*CI*	*Statistic*	*p*	*Estimates*	*CI*	*Statistic*	*p*
(Intercept)	-0.01	-0.03 – 0.01	-1.30	0.194	0.00	-0.01 – 0.02	0.60	0.548
result.e	0.37	0.31 – 0.42	13.39	**<0.001**	-0.39	-0.44 – -0.33	-13.83	**<0.001**
focus.e	-0.03	-0.05 – -0.00	-2.39	**0.017**	0.04	0.03 – 0.06	4.83	**<0.001**
agent.e	0.00	-0.01 – 0.01	0.05	0.961	-0.00	-0.01 – 0.01	-0.51	0.613
result.e * focus.e	-0.65	-0.76 – -0.54	-11.66	**<0.001**	0.68	0.57 – 0.80	11.79	**<0.001**
result.e * agent.e	-0.02	-0.04 – -0.00	-2.33	**0.020**	0.02	-0.01 – 0.04	1.45	0.146
focus.e * agent.e	-0.01	-0.03 – 0.01	-0.76	0.448	-0.01	-0.03 – 0.01	-0.92	0.357
(result.e * focus.e) * agent.e	-0.03	-0.07 – 0.01	-1.54	0.123	0.04	0.00 – 0.08	2.01	**0.044**
**Random Effects**								
σ^2	0.04				0.04			
τ_{00}	0.00 participant_id				0.00 participant_id			
	0.00 trial:GameRound							
	0.00 GameRound							
τ_{11}	0.06 participant_id.result.e				0.07 participant_id.result.e			
	0.01 participant_id.focus.e				0.01 participant_id.focus.e			
	0.26 participant_id.result.e:focus.e				0.28 participant_id.result.e:focus.e			
ρ_{01}	-0.16 participant_id.result.e				-0.10			
	-0.56 participant_id.focus.e				-0.29			
	0.04 participant_id.result.e:focus.e				-0.02			
ICC	0.56				0.55			
N	87 participant_id				87 participant_id			
	30 trial							
	3 GameRound							
Observations	7830				7830			
Marginal R^2 / Conditional R^2	0.461 / 0.762				0.485 / 0.768			

Table S8. Model summaries of LMEMs of ratingBad and ratingGood for experiment 3. (a) Estimated model of ratingBad (a) and rating good (b) fixed and random effects with 95% confidence intervals (CI) of experiment 3. The fixed effects are result (win, lose), focus (ingroup, outgroup) and agent type (human, robot). Intraclass correlation (ICC) depicts how much of the total variance is accounted for by clustering. The marginal R-squared value only considers the variance of the fixed effects, while the conditional R-squared takes both the fixed and random effects in account. Fitted model of both ratingGood and ratingBad is shown above the table.

Exploratory analyses - Team identification

Participants identified themselves more with their ingroup (before: 0.70 [0.67-0.74], after: 0.78 [0.74-0.82]) than their outgroup (before: 0.48 [0.43-0.53], after: 0.47 [0.42-0.52], β = -0.22, p<.001). Team identification changed over time (β = 0.08, p<0.001) and there was an interaction between time and group (β = -0.09, p=0.002). Team identification for their own team grew over time, while it remained stable for outgroup. This implies that arbitrarily assigning people to teams causes people to readily identify with their team which grows stronger over time.

(a)

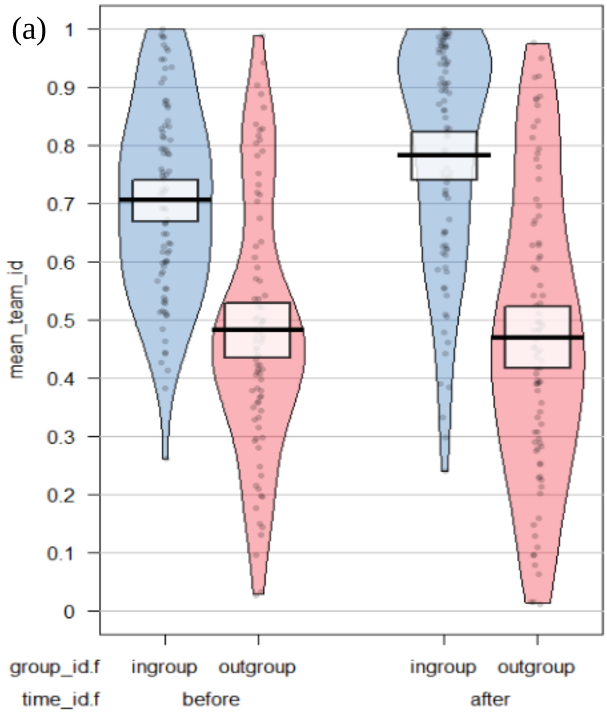

(b)

*mean_team_id ~ time_id * group_id*

	mean_team_id		
Predictors	*Estimates*	*CI*	*p*
(Intercept)	0.70	0.68 – 0.73	**<0.001**
time_id.f [after]	0.08	0.04 – 0.12	**<0.001**
group_id.f [outgroup]	-0.22	-0.26 – -0.18	**<0.001**
time_id.f [after] * group_id.f [outgroup]	-0.09	-0.15 – -0.03	**0.002**
Observations	1044		
R^2 / R^2 adjusted	0.255 / 0.253		

Figure S6. Team identification before and after the game in experiment 43 (a) Plot of team identification for the own team (blue) and opposing team (red) before and after the game in experiment 3. The dots represent the raw data and the beans the density of the responses. The black bar shows the mean with the white rectangle showing the 95% confidence interval. (b) Estimated model summary of multiple regression model with a fixed effect of time_id (before or after the game) and group_id (ingroup or outgroup team) with 95% confidence intervals (CI).

*Bad: rating ~ difid * result.e * focus.e * agent.e + (1 | GameRound/ trial)*
*Good: rating ~ difid * result.e * focus.e * agent.e*

	ratingsc				ratingsc			
Predictors	*Estimates*	*CI*	*Statistic*	*p*	*Estimates*	*CI*	*Statistic*	*p*
(Intercept)	-0.00	-0.01 – 0.01	-0.31	0.754	0.00	-0.01 – 0.01	0.23	0.818
difid	-0.04	-0.07 – -0.01	-3.00	**0.003**	0.01	-0.02 – 0.04	0.81	0.419
result.e	0.31	0.29 – 0.33	31.16	**<0.001**	-0.33	-0.35 – -0.31	-33.72	**<0.001**
focus.e	-0.03	-0.05 – -0.01	-2.61	**0.009**	0.04	0.03 – 0.06	4.54	**<0.001**
agent.e	-0.00	-0.02 – 0.02	-0.18	0.856	-0.00	-0.02 – 0.02	-0.37	0.715
difid * result.e	0.28	0.22 – 0.33	10.13	**<0.001**	-0.25	-0.30 – -0.19	-9.05	**<0.001**
difid * focus.e	0.00	-0.05 – 0.05	0.01	0.993	0.00	-0.05 – 0.05	0.02	0.986
result.e * focus.e	-0.57	-0.61 – -0.53	-28.82	**<0.001**	0.59	0.55 – 0.63	29.81	**<0.001**
difid * agent.e	0.01	-0.05 – 0.06	0.31	0.755	0.00	-0.05 – 0.06	0.14	0.889
result.e * agent.e	-0.02	-0.06 – 0.02	-1.15	0.249	0.02	-0.02 – 0.06	0.98	0.325
focus.e * agent.e	-0.01	-0.05 – 0.03	-0.56	0.574	-0.02	-0.06 – 0.02	-0.96	0.338
(difid * result.e) * focus.e	-0.37	-0.47 – -0.26	-6.68	**<0.001**	0.43	0.32 – 0.54	7.87	**<0.001**
(difid * result.e) * agent.e	-0.00	-0.11 – 0.10	-0.07	0.941	-0.03	-0.13 – 0.08	-0.47	0.640
(difid * focus.e) * agent.e	0.01	-0.10 – 0.12	0.18	0.859	0.04	-0.07 – 0.15	0.73	0.467
(result.e * focus.e) * agent.e	-0.04	-0.12 – 0.03	-1.12	0.264	0.05	-0.03 – 0.13	1.24	0.216
(difid * result.e * focus.e) * agent.e	0.03	-0.19 – 0.24	0.26	0.796	-0.01	-0.23 – 0.21	-0.09	0.928
**Random Effects**								
σ^2	0.09							
τ_{00}	0.00 trial:GameRound							
	0.00 GameRound							
ICC	0.00							
N	30 trial							
	3 GameRound							
Observations	7830				7830			
Marginal R^2 / Conditional R^2	0.481 / 0.483				0.502 / 0.501			

Table S9. Model summaries of LMEMs of ratingBad and ratingGood of experiment 3 when including difference in team identification. (a) Estimated model of ratingBad (a) and rating good (b) fixed and random effects with 95% confidence intervals (CI) of experiment 3 while including the difference in team identification (difid). Other fixed effects were result (win/lose), focus (ingroup/outgroup) and agent (human/robot). Intraclass correlation (ICC) depicts how much of the total variance is accounted for by clustering. The marginal R-squared value only considers the variance of the fixed effects, while the conditional R-squared takes both the fixed and random effects in account. Fitted model of both ratingGood and ratingBad is shown above the table.

Exploratory analyses – Blame

For experiment 3, the model on feeling bad showed an interaction effect between focus, agent type and blame ($\beta = 0.06$, $p=0.028$). Nevertheless, post-hoc tests did not point out a significant influence of blame on feeling bad. The model on feeling good showed an interaction effect between agent type and blame ($\beta = 0.03$, $p=0.021$). Participants felt better about an outgroup human who was blamed for losing (0.37 [0.31,0.44]) rather than an outgroup human who was not blamed (0.32 [0.26,0.39], $p=0.028$). These results indicate that people felt more schadenfreude towards human outgroup members when they were to be blamed for losing.

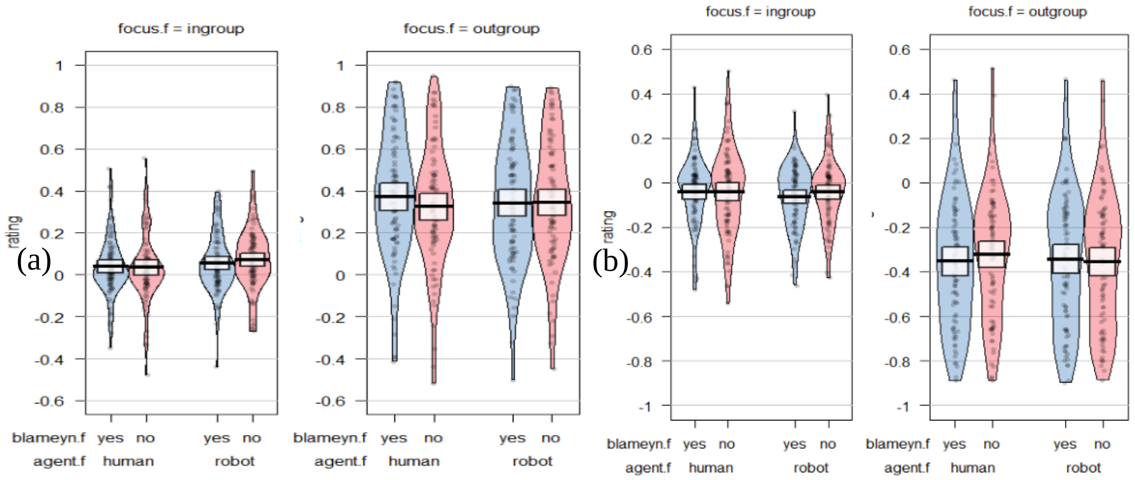

Figure S7. Visualization of relation between blame and rating in experiment 3. Plot of the effect of blame, with yes (this person is the one to blame for losing) in blue and no (this person is not the one to blame for losing) in red, for agent type (human/robot) and focus (ingroup/outgroup) on ratingGood (a) and ratingBad (b) when losing. The dots represent the raw data and the beans the density of the responses. The black bar shows the mean with the white rectangle showing the 95% confidence interval.

*rating ~ focus.e * agent.e * blameyn.e + (1 + focus.e * blameyn.e | participant_id) + (1 | GameRound/trial)*

	**ratingsc**				**ratingsc**			
Predictors	*Estimates*	*CI*	*Statistic*	*p*	*Estimates*	*CI*	*Statistic*	*p*
(Intercept)	-0.19	-0.24 – -0.15	-9.11	**<0.001**	0.20	0.16 – 0.23	11.54	**<0.001**
focus.e	0.30	0.24 – 0.36	9.58	**<0.001**	-0.30	-0.36 – -0.24	-9.41	**<0.001**
agent.e	0.02	0.00 – 0.03	2.15	**0.031**	-0.01	-0.02 – 0.00	-1.40	0.160
blameyn.e	-0.01	-0.03 – 0.01	-1.02	0.307	0.01	-0.01 – 0.02	0.83	0.406
focus.e * agent.e	0.03	-0.00 – 0.06	1.75	0.080	-0.03	-0.06 – -0.00	-2.18	**0.029**
focus.e * blameyn.e	-0.00	-0.06 – 0.05	-0.02	0.988	-0.03	-0.09 – 0.04	-0.83	0.405
agent.e * blameyn.e	-0.01	-0.04 – 0.02	-0.60	0.550	0.03	0.01 – 0.06	2.32	**0.021**
(focus.e * agent.e) * blameyn.e	0.06	0.01 – 0.12	2.19	**0.028**	-0.02	-0.08 – 0.03	-0.78	0.435
**Random Effects**								
σ^2	0.04				0.04			
τ_{00}	0.02 $_{participant\_id}$				0.02 $_{participant\_id}$			
	0.00 $_{trial:GameRound}$				0.00 $_{trial:GameRound}$			
	0.00 $_{GameRound}$				0.00 $_{GameRound}$			
τ_{11}	0.08 $_{participant\_id.focus.e}$				0.08 $_{participant\_id.focus.e}$			
	0.00 $_{participant\_id.blameyn.e}$				0.00 $_{participant\_id.blameyn.e}$			
	0.05 $_{participant\_id.focus.e:blameyn.e}$				0.08 $_{participant\_id.focus.e:blameyn.e}$			
ρ_{01}	-0.84 $_{participant\_id.focus.e}$				-0.87 $_{participant\_id.focus.e}$			
	0.18 $_{participant\_id.blameyn.e}$				0.02 $_{participant\_id.blameyn.e}$			
	-0.06 $_{participant\_id.focus.e:blameyn.e}$				-0.04 $_{participant\_id.focus.e:blameyn.e}$			
ICC	0.64				0.64			
N	87 $_{participant\_id}$				87 $_{participant\_id}$			
	30 $_{trial}$				30 $_{trial}$			
	3 $_{GameRound}$				3 $_{GameRound}$			
Observations	4176				4176			
Marginal R^2 / Conditional R^2	0.140 / 0.693				0.138 / 0.694			

Table S10. Model summaries of LMEMs of ratingBad and ratingGood when including blame for Experiment 3. (a) Estimated model of ratingBad (a) and rating good (b) fixed and random effects with 95% confidence intervals (CI) of Experiment 3 while including blame (blameyn.e). Other fixed effects were focus (ingroup/ outgroup) and agent (human/robot). Result was not included as fixed effect, as one could only be blamed when their team lost the game. Intraclass correlation (ICC) depicts how much of the total variance is accounted for by clustering. The marginal R-squared value only considers the variance of the fixed effects, while the conditional R-squared takes both the fixed and random effects in account. Fitted model of both ratingGood and ratingBad is shown above the table.

Exploratory analyses – Difference scores

In experiment 3, the findings of the model for feeling bad showed that there was a main effect of the difference scores (β = -0.14, $p<.001$). When the difference scores increased, people tended to feel less bad for losing. The model for feeling good also showed a main effect of the difference scores (β = 0.08, $p=0.014$). When the difference scores increased, people also tended to feel better when winning and losing. These results indicate that people had a better overall mood when the difference in scores was high and to the benefit of the participant.

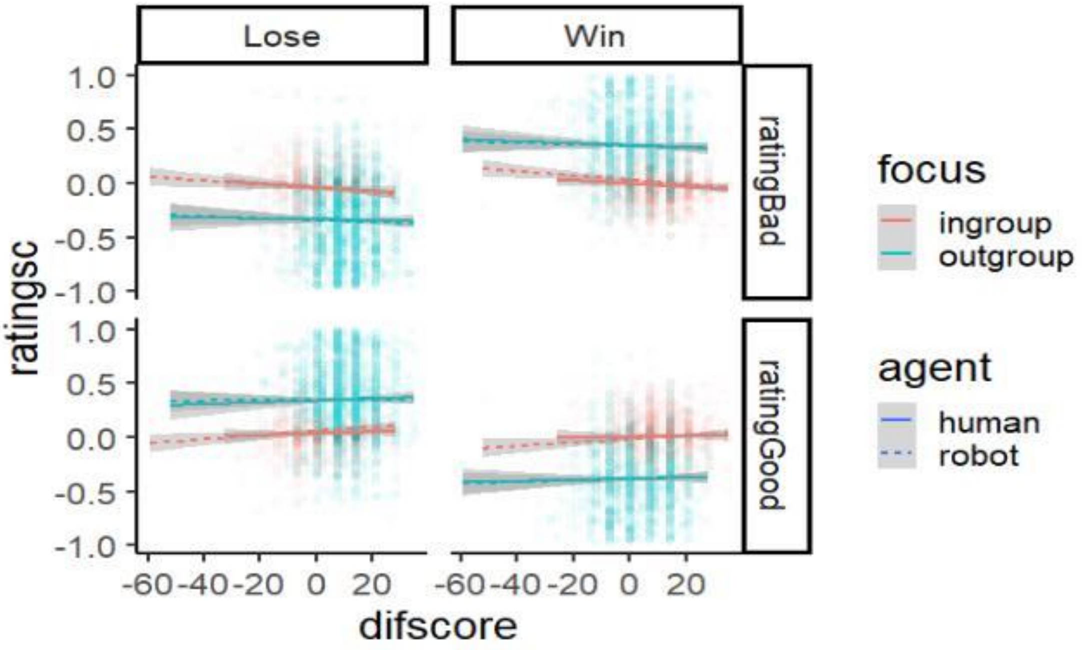

Figure S8. Visualization of relation between rating and the difference in scores in Experiment 3. Plot shows the raw data and linear regression lines of the fitted models with 95% confidence intervals depicting the relation between rating and the difference in scores. This is shown for all given conditions: result (Win/Lose), focus (ingroup/outgroup), agent type (human/robot) and the valence of the question (ratingBad/ratingGood).

*Bad: rating ~ difscore.s * result.e * focus.e * agent.e + (1 + result.e * focus.e | participant_id)*
*Good: rating ~ difscore.s*result.e*focus.e*agent.e+(1+result.e+focus.e|participant_id)+(1| GameRound/trial).*

	**ratingsc**				**ratingsc**			
Predictors	*Estimates*	*CI*	*Statistic*	*p*	*Estimates*	*CI*	*Statistic*	*p*
(Intercept)	-0.00	-0.02 – 0.01	-0.33	0.739	-0.00	-0.02 – 0.02	-0.07	0.948
difscore.s	-0.14	-0.20 – -0.09	-5.27	**<0.001**	0.08	0.02 – 0.15	2.47	**0.014**
result.e	0.37	0.31 – 0.42	13.31	**<0.001**	-0.39	-0.46 – -0.31	-10.37	**<0.001**
focus.e	-0.02	-0.05 – -0.00	-2.04	**0.041**	0.04	0.02 – 0.06	4.34	**<0.001**
agent.e	-0.00	-0.01 – 0.01	-0.27	0.784	-0.00	-0.02 – 0.01	-0.26	0.791
difscore.s * result.e	0.02	-0.08 – 0.13	0.45	0.652	-0.05	-0.18 – 0.07	-0.83	0.408
difscore.s * focus.e	-0.08	-0.18 – 0.03	-1.42	0.155	0.05	-0.07 – 0.18	0.84	0.402
result.e * focus.e	-0.62	-0.73 – -0.51	-11.13	**<0.001**	0.67	0.65 – 0.70	45.18	**<0.001**
difscore.s * agent.e	0.00	-0.10 – 0.11	0.09	0.928	0.00	-0.12 – 0.13	0.04	0.964
result.e * agent.e	-0.03	-0.05 – -0.01	-2.35	**0.019**	0.02	-0.01 – 0.05	1.60	0.109
focus.e * agent.e	-0.02	-0.05 – 0.00	-1.81	0.070	0.01	-0.02 – 0.04	0.38	0.701
(difscore.s * result.e) * focus.e	-0.05	-0.26 – 0.17	-0.42	0.674	-0.05	-0.29 – 0.19	-0.40	0.691
(difscore.s * result.e) * agent.e	-0.02	-0.23 – 0.19	-0.20	0.838	-0.08	-0.32 – 0.16	-0.63	0.531
(difscore.s * focus.e) * agent.e	0.16	-0.05 – 0.38	1.51	0.132	-0.14	-0.39 – 0.11	-1.07	0.284
(result.e * focus.e) * agent.e	-0.04	-0.09 – 0.01	-1.54	0.123	0.04	-0.02 – 0.10	1.42	0.155
[illegible]	[illegible]	[illegible]	[illegible]	[illegible]	[illegible]	[illegible]	[illegible]	[illegible]

**Random Effects**		
σ^2	0.04	0.06
τ_{00}	0.00 participant_id	0.00 participant_id
		0.00 trial:GameRound
		0.00 GameRound
τ_{11}	0.06 participant_id.result.e	0.12 participant_id.result.e
	0.01 participant_id.focus.e	0.00 participant_id.focus.e
	0.26 participant_id.result.e:focus.e	
ρ_{01}	-0.17	-0.53 participant_id.result.e
	-0.56	-0.30 participant_id.focus.e
	0.04	
ICC	0.56	0.38
N	87 participant_id	87 participant_id
		30 trial
		3 GameRound
Observations	7830	7830
Marginal R^2 / Conditional R^2	0.462 / 0.761	0.486 / 0.682

Table

Table S11. Model summaries of LMEMs of ratingBad and ratingGood when including the difference in scores. (a) Estimated model of ratingBad (left) and rating good (right) fixed and random effects with 95% confidence intervals (CI) of experiment 3 while including the difference in scores as fixed-effect (difscore). Other fixed effects were result (win/lose), focus (ingroup/outgroup) and agent (human/robot). Intraclass correlation (ICC) depicts how much of the total variance is accounted for by clustering. The marginal R-squared value only considers the variance of the fixed effects, while the conditional R-squared takes both the fixed and random effects in account. Fitted model of both ratingGood and ratingBad is shown above the table.

Experiment 4
Main analyses

*rating ~ result.e * focus.e * agent.e + (1 | participant_id) + (0 + result.e * focus.e | participant_id) + (1 | GameRound/trial)*

	ratingsc				ratingsc			
Predictors	*Estimates*	*CI*	*Statistic*	*p*	*Estimates*	*CI*	*Statistic*	*p*
(Intercept)	0.00	-0.02 – 0.03	0.34	0.736	-0.01	-0.02 – 0.01	-1.04	0.296
result.e	0.41	0.36 – 0.47	14.19	**<0.001**	-0.47	-0.52 – -0.41	-17.16	**<0.001**
focus.e	-0.01	-0.03 – 0.00	-1.44	0.151	0.03	0.01 – 0.05	3.56	**<0.001**
agent.e	-0.00	-0.01 – 0.01	-0.57	0.569	0.00	-0.01 – 0.01	0.63	0.530
result.e * focus.e	-0.76	-0.87 – -0.65	-13.66	**<0.001**	0.86	0.75 – 0.96	15.40	**<0.001**
result.e * agent.e	-0.02	-0.04 – -0.00	-2.32	**0.021**	0.03	0.01 – 0.05	3.42	**0.001**
focus.e * agent.e	0.01	-0.01 – 0.03	1.37	0.171	-0.02	-0.04 – 0.00	-1.59	0.113
(result.e * focus.e) * agent.e	-0.02	-0.05 – 0.02	-0.81	0.417	0.01	-0.02 – 0.05	0.74	0.462
N	93 $_{\text{participant\_id}}$				93 $_{\text{participant\_id}}$			
	30 $_{\text{trial}}$				30 $_{\text{trial}}$			
	3 $_{\text{GameRound}}$				3 $_{\text{GameRound}}$			
Observations	8370				8370			

Table S12. Model summaries of LMEMs of ratingBad and ratingGood for experiment 4. (a) Estimated model of ratingBad (a) and rating good (b) fixed and random effects with 95% confidence intervals (CI) of experiment 4. The fixed effects are result (win, lose), focus (ingroup, outgroup) and agent type (human, robot). Intraclass correlation (ICC) depicts how much of the total variance is accounted for by clustering. The marginal R-squared value only considers the variance of the fixed effects, while the conditional R-squared takes both the fixed and random effects in account. Fitted model of both ratingGood and ratingBad is shown above the table.

Exploratory analyses – Team identification

Participants identified themselves more with their ingroup (before: 0.72 [0.68-0.75], after: 0.80 [0.76-0.83]) than their outgroup (before: 0.46 [0.41-0.50], after: 0.44 [0.39-0.49], β = -0.26, p<.001). However, team identification changed over time (β = 0.08, p<0.001) and there was an interaction between time and group (β = -0.10, p<0.001; see Figure S11a). Participants identified themselves more with their own team over time, while it remained stable for the outgroup team. This suggests that arbitrarily assigning people to teams causes people to readily identify with their team which grows stronger over time.

(a)

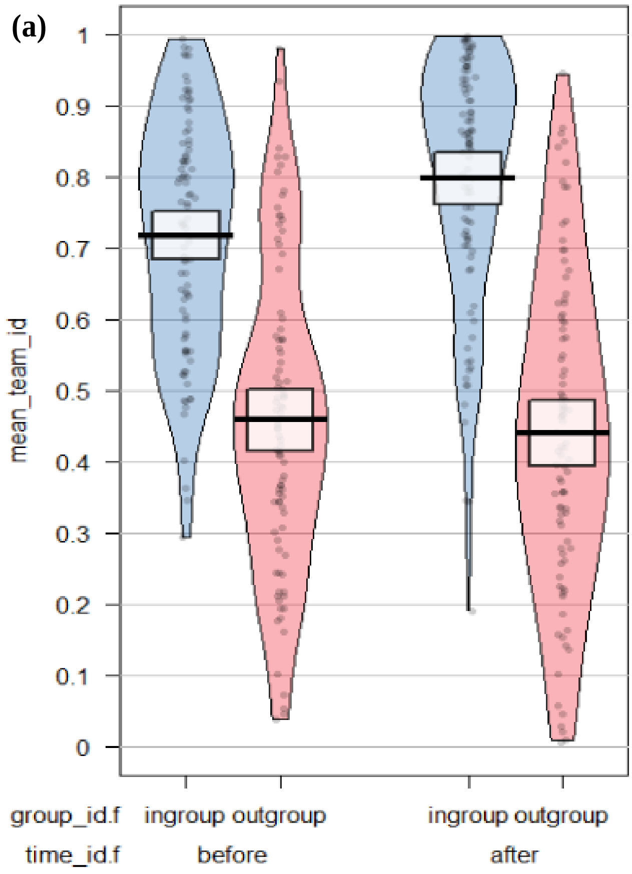

(b)

*mean_team_id ~ time_id * group_id*

	mean_team_id		
Predictors	*Estimates*	*CI*	*p*
(Intercept)	0.72	0.69 – 0.74	**<0.001**
time_id.f [after]	0.08	0.04 – 0.12	**<0.001**
group_id.f [outgroup]	-0.26	-0.30 – -0.22	**<0.001**
time_id.f [after] * group_id.f [outgroup]	-0.10	-0.15 – -0.05	**<0.001**
Observations	1116		
R^2 / R^2 adjusted	0.335 / 0.334		

Figure S9. Team identification before and after the game in experiment 4 (a) Plot of team identification for the own team (blue) and opposing team (red) before and after the game in experiment 4. The dots represent the raw data and the beans the density of the responses. The black bar shows the mean with the white rectangle showing the 95% confidence interval. (b) Estimated model summary of multiple regression model with a fixed effect of time_id (before or after the game) and group_id (ingroup or outgroup team) with 95% confidence intervals (CI).

*rating ~ difid * result.e * focus.e * agent.e + (1 | GameRound/trial)*

	ratingsc				ratingsc			
Predictors	*Estimates*	*CI*	*Statistic*	*p*	*Estimates*	*CI*	*Statistic*	*p*
(Intercept)	0.00	-0.02 – 0.02	0.01	0.995	-0.01	-0.03 – 0.00	-1.36	0.174
difid	0.01	-0.01 – 0.04	1.20	0.231	0.01	-0.02 – 0.03	0.69	0.488
result.e	0.34	0.32 – 0.36	34.48	**<0.001**	-0.39	-0.41 – -0.38	-41.52	**<0.001**
focus.e	-0.02	-0.04 – 0.00	-1.91	0.056	0.04	0.02 – 0.06	4.34	**<0.001**
agent.e	-0.01	-0.03 – 0.01	-0.89	0.375	0.01	-0.01 – 0.03	0.88	0.378
difid * result.e	0.31	0.26 – 0.36	12.54	**<0.001**	-0.28	-0.33 – -0.23	-11.76	**<0.001**
difid * focus.e	0.03	-0.02 – 0.08	1.11	0.269	-0.04	-0.09 – 0.00	-1.83	0.068
result.e * focus.e	-0.55	-0.59 – -0.51	-28.09	**<0.001**	0.65	0.61 – 0.69	34.03	**<0.001**
difid * agent.e	0.02	-0.03 – 0.07	0.77	0.444	-0.02	-0.06 – 0.03	-0.72	0.471
result.e * agent.e	-0.01	-0.05 – 0.03	-0.48	0.633	0.03	-0.01 – 0.07	1.69	0.091
focus.e * agent.e	0.00	-0.04 – 0.04	0.12	0.905	-0.01	-0.05 – 0.03	-0.59	0.557
(difid * result.e) * focus.e	-0.82	-0.92 – -0.72	-16.65	**<0.001**	0.80	0.71 – 0.90	16.79	**<0.001**
(difid * result.e) * agent.e	-0.00	-0.10 – 0.09	-0.08	0.940	-0.04	-0.13 – 0.05	-0.83	0.408
(difid * focus.e) * agent.e	0.04	-0.06 – 0.13	0.71	0.478	-0.02	-0.11 – 0.08	-0.38	0.702
(result.e * focus.e) * agent.e	0.01	-0.07 – 0.09	0.24	0.813	0.01	-0.07 – 0.08	0.20	0.844
(difid * result.e * focus.e) * agent.e	-0.15	-0.34 – 0.04	-1.50	0.132	0.07	-0.11 – 0.26	0.77	0.440
**Random Effects**								
σ^2	0.09				0.08			
τ_{00}	0.00 $_{\text{trial:GameRound}}$				0.00 $_{\text{trial:GameRound}}$			
	0.00 $_{\text{GameRound}}$				0.00 $_{\text{GameRound}}$			
ICC	0.01				0.01			
N	30 $_{\text{trial}}$				30 $_{\text{trial}}$			
	3 $_{\text{GameRound}}$				3 $_{\text{GameRound}}$			
Observations	8370				8370			
Marginal R^2 / Conditional R^2	0.562 / 0.567				0.628 / 0.631			

Table S13. Model summaries of LMEMs of ratingBad and ratingGood of experiment 4 when including difference in team identification. Estimated model of ratingBad (left) and rating good (right) fixed and random effects with 95% confidence intervals (CI) of experiment 4 while including the difference in team identification (difid). Other fixed effects were result (win/lose), focus (ingroup/outgroup) and agent (human/robot). Intraclass correlation (ICC) depicts how much of the total variance is accounted for by clustering. The marginal R-squared value only considers the variance of the fixed effects, while the conditional R-squared takes both the fixed and random effects in account. Fitted model of both ratingGood and ratingBad is shown above the table.

Exploratory analyses – Blame

Both the model on feeling bad and the model on feeling good in experiment 4 did not show an influence of blame on how participants rated their feelings. People therefore do not seem to feel more or less empathy or schadenfreude for someone when that individual is blamed for letting their team lose.

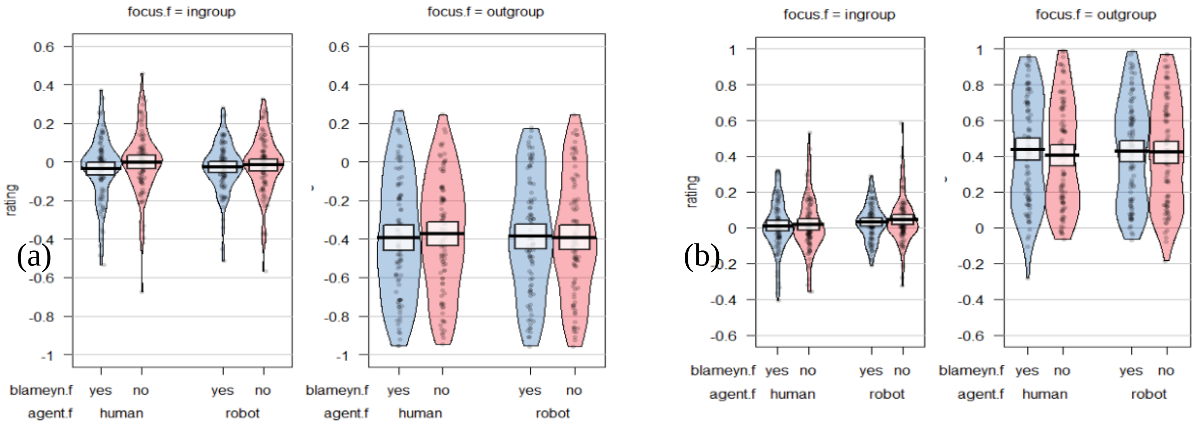

Figure S10. Visualization of relation between blame and rating in experiment 4. Plot of the effect of blame, with yes (this person is the one to blame for losing) in blue and no (this person is not the one to blame for losing) in red, for agent type (human/robot) and focus (ingroup/outgroup) on feeling good (a) and feeling bad (b) when losing. The dots represent the raw data and the beans the density of the responses. The black bar shows the mean with the white rectangle showing the 95% confidence interval.

*rating ~ focus.e * agent.e * blameyn.e + (1 + focus.e * blameyn.e | participant_id) + (1 | GameRound/trial)*

	ratingsc				ratingsc			
Predictors	*Estimates*	*CI*	*Statistic*	*p*	*Estimates*	*CI*	*Statistic*	*p*
(Intercept)	-0.20	-0.27 – -0.14	-6.10	**<0.001**	0.22	0.18 – 0.27	9.92	**<0.001**
focus.e	0.38	0.32 – 0.44	12.34	**<0.001**	-0.40	-0.46 – -0.34	-13.20	**<0.001**
agent.e	0.02	0.00 – 0.03	2.24	**0.025**	-0.02	-0.03 – -0.01	-2.79	**0.005**
blameyn.e	-0.01	-0.03 – 0.00	-1.60	0.110	0.00	-0.01 – 0.02	0.59	0.557
focus.e * agent.e	0.06	0.03 – 0.09	4.30	**<0.001**	-0.06	-0.09 – -0.03	-3.83	**<0.001**
focus.e * blameyn.e	-0.02	-0.06 – 0.03	-0.66	0.507	-0.03	-0.08 – 0.01	-1.32	0.186
agent.e * blameyn.e	-0.02	-0.05 – 0.00	-1.69	0.090	0.02	-0.01 – 0.05	1.30	0.195
(focus.e * agent.e) * blameyn.e	0.00	-0.05 – 0.05	0.04	0.970	-0.02	-0.08 – 0.03	-0.83	0.406
**Random Effects**								
σ^2	0.03				0.04			
τ_{00}	0.03 $_{participant\_id}$				0.02 $_{participant\_id}$			
	0.00 $_{trial:GameRound}$				0.00 $_{trial:GameRound}$			
	0.00 $_{GameRound}$				0.00 $_{GameRound}$			
τ_{11}	0.08 $_{participant\_id.focus.e}$				0.08 $_{participant\_id.focus.e}$			
	0.00 $_{participant\_id.blameyn.e}$				0.00 $_{participant\_id.blameyn.e}$			
	0.03 $_{participant\_id.focus.e:blameyn.e}$				0.03 $_{participant\_id.focus.e:blameyn.e}$			
ρ_{01}	-0.89 $_{participant\_id.focus.e}$				-0.94 $_{participant\_id.focus.e}$			
	0.09 $_{participant\_id.blameyn.e}$				0.04 $_{participant\_id.blameyn.e}$			
	0.02 $_{participant\_id.focus.e:blameyn.e}$				-0.08 $_{participant\_id.focus.e:blameyn.e}$			
ICC	0.68				0.64			
N	93 $_{participant\_id}$				93 $_{participant\_id}$			
	30 $_{trial}$				30 $_{trial}$			
	3 $_{GameRound}$				3 $_{GameRound}$			
Observations	4464				4464			
Marginal R^2 / Conditional R^2	0.192 / 0.739				0.227 / 0.718			

Table S14. Model summaries of LMEMs of ratingBad and ratingGood when including blame for Experiment 4. (a) Estimated model of ratingBad (left) and rating good (right) fixed and random effects with 95% confidence intervals (CI) of experiment 4 while including blame (blameyn.e). Other fixed effects were focus (ingroup/ outgroup) and agent (human/robot). Result was not included as fixed effect, as one could only be blamed when their team lost the game. Intraclass correlation (ICC) depicts how much of the total variance is accounted for by clustering. The marginal R-squared value only considers the variance of the fixed effects, while the conditional R-squared takes both the fixed and random effects in account. Fitted model of both ratingGood and ratingBad is shown above the table.

Exploratory analyses – Difference scores

For experiment 4, the findings of the model for feeling bad showed that there was a main effect of the difference scores (β = -0.12, p<.001) and an interaction effect between the difference scores and focus (β = -0.24, p=0.016). Participants felt less bad about losing for both ingroup and outgroup members in a similar way though when the difference in scores increases. The model for feeling good showed a main effect of the difference scores as well (β = 0.11, p<.001), just like an interaction effect between the difference scores and focus (β =-0.33, p=0.001). When the difference in scores increased, participants felt better for both winning and losing, but especially when ingroup members won.

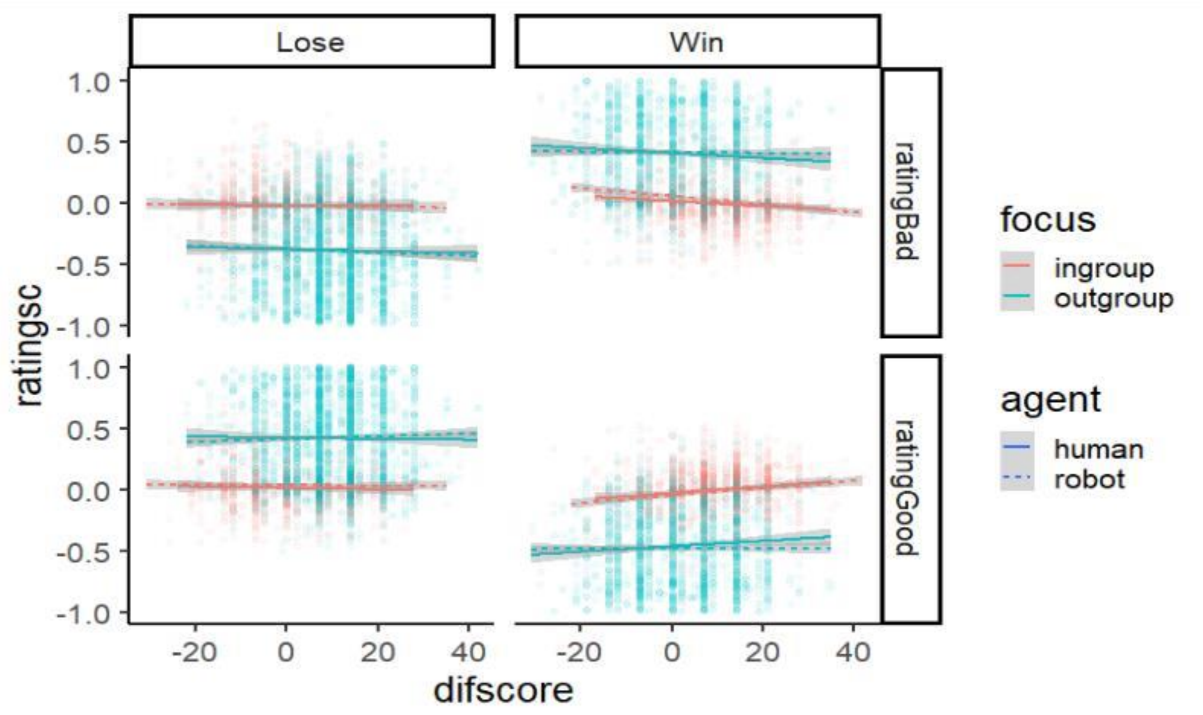

Figure S11. Visualization of relation between rating and the difference in scores in Experiment 4. Plot shows the raw data and linear regression lines of the fitted models with 95% confidence intervals depicting the relation between rating and the difference in scores in experiment 4. This is shown for all given conditions: result (Win/Lose), focus (ingroup/outgroup), agent type (human/robot) and the valence of the question (ratingBad/ratingGood).

*Bad: rating ~ difscore.s * result.e * focus.e * agent.e + (1 | participant_id) + (0 + result.e * focus.e | participant_id) + (1 | GameRound/trial)*
*Good: rating ~ difid * result.e * focus.e * agent.e + (1 | participant_id) + (0 + result.e * focus.e | participant_id)*

	**ratingsc**				**ratingsc**			
Predictors	*Estimates*	*CI*	*Statistic*	*p*	*Estimates*	*CI*	*Statistic*	*p*
(Intercept)	0.01	-0.01 – 0.04	1.04	0.298	-0.02	-0.03 – -0.01	-3.05	**0.002**
difscore.s	-0.12	-0.18 – -0.07	-4.65	**<0.001**	0.11	0.06 – 0.15	4.27	**<0.001**
result.e	0.42	0.36 – 0.48	14.36	**<0.001**	-0.47	-0.53 – -0.42	-17.37	**<0.001**
focus.e	-0.00	-0.02 – 0.01	-0.52	0.606	0.02	0.01 – 0.04	2.54	**0.011**
agent.e	-0.01	-0.02 – 0.01	-0.99	0.320	0.01	-0.00 – 0.02	1.28	0.202
difscore.s * result.e	-0.08	-0.17 – 0.02	-1.55	0.120	0.11	0.01 – 0.20	2.17	**0.030**
difscore.s * focus.e	-0.08	-0.18 – 0.02	-1.65	0.099	0.07	-0.02 – 0.17	1.47	0.141
result.e * focus.e	-0.73	-0.84 – -0.62	-13.04	**<0.001**	0.82	0.71 – 0.93	14.71	**<0.001**
difscore.s * agent.e	0.02	-0.07 – 0.11	0.41	0.684	-0.02	-0.11 – 0.08	-0.39	0.696
result.e * agent.e	-0.03	-0.05 – -0.01	-2.63	**0.009**	0.03	0.01 – 0.06	2.98	**0.003**
focus.e * agent.e	0.00	-0.02 – 0.03	0.34	0.735	-0.01	-0.03 – 0.01	-0.81	0.418
(difscore.s * result.e) * focus.e	-0.24	-0.43 – -0.04	-2.40	**0.016**	0.33	0.14 – 0.52	3.38	**0.001**
(difscore.s * result.e) * agent.e	0.07	-0.12 – 0.26	0.70	0.481	0.09	-0.10 – 0.28	0.95	0.341
(difscore.s * focus.e) * agent.e	0.10	-0.09 – 0.29	1.02	0.309	-0.13	-0.32 – 0.06	-1.30	0.194
(result.e * focus.e) * agent.e	-0.03	-0.07 – 0.02	-1.19	0.234	0.03	-0.01 – 0.08	1.47	0.142
(difscore.s * result.e * focus.e) * agent.e	0.15	-0.22 – 0.53	0.80	0.425	-0.29	-0.66 – 0.09	-1.49	0.135
**Random Effects**								
σ^2					0.04			
τ_{00}					0.00 participant_id			
					0.07 participant_id.1			
τ_{11}					0.00 participant_id.1.focus.e			
					0.28 participant_id.1.result.e:focus.e			
ρ_{01}					0.35 participant_id.1.focus.e			
					-0.97 participant_id.1.result.e:focus.e			
ICC					0.05			
N	93 participant_id				93 participant_id			
	30 trial							
	3 GameRound							
Observations	8370				8370			
Marginal R^2 / Conditional R^2	NA				0.762 / 0.775			

Table S15. Model summaries of LMEMs of ratingBad and ratingGood when including the difference in scores in Experiment 4. (a) Estimated model of ratingBad (left) and rating good (right) fixed and random effects with 95% confidence intervals (CI) of experiment 3 while including the difference in scores as fixed-effect (difscore). Other fixed effects were result (win/lose), focus (ingroup/outgroup) and agent (human/robot). Intraclass correlation (ICC) depicts how much of the total variance is accounted for by clustering. The marginal R-squared value only considers the variance of the fixed effects, while the conditional R-squared takes both the fixed and random effects in account. Fitted model of both ratingGood and ratingBad is shown above the table.

Experiment 3 and 4 merged

	ratingsc				ratingsc			
Predictors	*Estimates*	*CI*	*Statistic*	*p*	*Estimates*	*CI*	*Statistic*	*p*
(Intercept)	-0.00	-0.02 – 0.01	-0.33	0.742	-0.00	-0.01 – 0.01	-0.42	0.676
result.e	0.40	0.36 – 0.44	19.70	**<0.001**	-0.43	-0.47 – -0.39	-22.05	**<0.001**
focus.e	-0.02	-0.03 – -0.01	-2.96	**0.003**	0.04	0.03 – 0.05	6.55	**<0.001**
agent.f1	0.01	-0.00 – 0.02	1.69	0.091	-0.01	-0.02 – 0.00	-1.74	0.083
agent.f2	-0.00	-0.01 – 0.01	-0.31	0.753	0.00	-0.01 – 0.01	0.23	0.815
result.e * focus.e	-0.70	-0.78 – -0.63	-17.85	**<0.001**	0.77	0.69 – 0.85	19.07	**<0.001**
result.e : agent.f1	-0.02	-0.04 – 0.01	-1.37	0.171	-0.00	-0.03 – 0.02	-0.08	0.936
result.e : agent.f2	-0.03	-0.05 – -0.01	-3.28	**0.001**	0.03	0.01 – 0.05	3.35	**0.001**
focus.e : agent.f1	0.01	-0.02 – 0.03	0.56	0.575	-0.01	-0.03 – 0.01	-0.84	0.402
focus.e : agent.f2	0.00	-0.02 – 0.02	0.45	0.653	-0.01	-0.03 – 0.01	-1.18	0.239
result.e : focus.e : agent.f1	-0.04	-0.09 – 0.01	-1.46	0.145	0.06	0.01 – 0.11	2.24	**0.025**
result.e : focus.e : agent.f2	-0.03	-0.07 – 0.01	-1.68	0.092	0.04	0.00 – 0.08	1.97	**0.049**

Table S16. Model summaries of LMEMs of ratingBad and ratingGood for experiment 3 and 4. (a) Estimated model of ratingBad (a) and rating good (b) fixed and random effects with 95% confidence intervals (CI) of experiment 4. The fixed effects included result (win, lose) and focus (ingroup, outgroup) and agent (Cozmo, NAO, human). Agent.f1 compared the Cozmo with the NAO, while agent.f2 compared the NAO with the human. Intraclass correlation (ICC) depicts how much of the total variance is accounted for by clustering. The marginal R-squared value only considers the variance of the fixed effects, while the conditional R-squared takes both the fixed and random effects in account. Fitted model of both ratingGood and ratingBad is shown above the table.

Exploratory analyses regarding human- and robot-believers

Further analyses show that the participants most often described the other human player as a human (N_{Exp3}=61; N_{Exp4}=51), rather than a bot (N_{Exp3}=2; N_{Exp4}=3) or undefined (N_{Exp3}=28; N_{Exp4}=34). Participants also described the two robot players most often as a human (N_{Exp3}=76; N_{Exp4}=81), rather than a bot (N_{Exp3}=38; N_{Exp4}=37) or undefined (N_{Exp3}=43; N_{Exp4}=41). Even after introducing participants to the robot, they still described the two robot players as a human in just a little less than half of the time. The following results show the results of LMEMs divided by whether people who believed they played against a robot or whether they believed to have played with another human.

	ratingsc				ratingsc			
Predictors	*Estimates*	*CI*	*Statistic*	*p*	*Estimates*	*CI*	*Statistic*	*p*
(Intercept)	-0.01	-0.03 – 0.01	-0.90	0.369	-0.01	-0.02 – 0.01	-0.93	0.350
result.e	0.44	0.36 – 0.52	10.96	**<0.001**	-0.48	-0.57 – -0.39	-10.12	**<0.001**
focus.e	-0.01	-0.04 – 0.02	-0.65	0.518	0.03	0.00 – 0.05	2.14	**0.032**
agent.f1	0.01	-0.02 – 0.03	0.66	0.507	-0.02	-0.05 – 0.00	-1.64	0.100
agent.f2	-0.00	-0.02 – 0.02	-0.16	0.876	-0.01	-0.03 – 0.01	-0.60	0.548
result.e * focus.e	-0.75	-0.90 – -0.61	-10.14	**<0.001**	0.81	0.78 – 0.84	52.97	**<0.001**
result.e * agent.f1	-0.01	-0.06 – 0.04	-0.39	0.696	-0.01	-0.08 – 0.05	-0.30	0.761
result.e * agent.f2	-0.04	-0.07 – -0.00	-2.09	**0.036**	0.04	-0.00 – 0.08	1.85	0.064
focus.e * agent.f1	0.01	-0.04 – 0.06	0.45	0.650	-0.08	-0.13 – -0.03	-2.99	**0.003**
focus.e * agent.f2	-0.02	-0.06 – 0.02	-1.11	0.269	-0.05	-0.09 – -0.01	-2.29	**0.022**
result.e * focus.e * agent.f1	-0.12	-0.22 – -0.03	-2.47	**0.013**	0.48	0.39 – 0.57	10.77	**<0.001**
result.e * focus.e * agent.f2	-0.06	-0.13 – 0.01	-1.61	0.108	0.10	0.01 – 0.18	2.25	**0.025**

Table S17. Model summaries of LMEMs of ratingBad and ratingGood for experiment 3 and 4 where participants believed they played against a robot. Estimated model of ratingBad (left) and ratingGood (right) fixed effects with 95% confidence intervals (CI) of experiments 3 and 4 when people thought they played against robots. The effects included result (win, lose) and focus (ingroup, outgroup) and agent (Cozmo, NAO, human). Agent.f1 compared the Cozmo with the NAO, while agent.f2 compared the NAO with the human. The p-value indicated if the fixed effect had a significant effect on the ratings.

Table S18. Posthoc analyses – ratingGood for those who believed to play against robots.

	Contrast	Estimate	SE	df	t.ratio	p.value
**Losing outgroup**						
	Cozmo - NAO	-0.13	0.02	2426	-6.06	<.001
	Cozmo – human	-0.08	0.02	3560	-4.84	<.001
	NAO - human	0.05	0.02	3804	3.22	.004
**Winning outgroup**						
	Cozmo - NAO	0.12	0.02	1032	4.76	<.001
	Cozmo – human	0.05	0.02	2583	2.39	.045
	NAO - human	-0.07	0.02	3174	-3.83	<.001
**Losing ingroup**						
	Cozmo - NAO	0.14	0.03	738	4.83	<.001
	Cozmo – human	0.13	0.03	2574	4.84	<.001
	NAO - human	-0.01	0.02	3234	-0.84	.840
**Winning ingroup**						
	Cozmo - NAO	-0.05	0.02	453	-1.92	.135
	Cozmo – human	-0.03	0.02	1941	-1.53	.276
	NAO - human	0.01	0.02	2708	0.67	.779

Degrees-of-freedom method: kenward-roger

P value adjustment: tukey method for comparing a family of 3 estimates

	ratingsc				ratingsc			
Predictors	*Estimates*	*CI*	*Statistic*	*p*	*Estimates*	*CI*	*Statistic*	*p*
(Intercept)	-0.01	-0.04 – 0.02	-0.66	0.508	0.00	-0.01 – 0.02	0.42	0.675
result.e	0.42	0.36 – 0.48	14.43	**<0.001**	-0.45	-0.50 – -0.39	-15.49	**<0.001**
focus.e	-0.02	-0.03 – -0.00	-2.03	**0.042**	0.04	0.02 – 0.05	4.30	**<0.001**
agent.f1	0.01	-0.01 – 0.03	0.83	0.409	-0.01	-0.03 – 0.01	-0.88	0.377
agent.f2	-0.00	-0.01 – 0.01	-0.09	0.929	0.00	-0.01 – 0.02	0.45	0.650
result.e * focus.e	-0.74	-0.85 – -0.62	-12.82	**<0.001**	0.80	0.68 – 0.92	13.23	**<0.001**
result.e * agent.f1	-0.04	-0.08 – -0.00	-2.14	**0.033**	0.02	-0.01 – 0.06	1.37	0.172
result.e * agent.f2	-0.03	-0.06 – -0.01	-2.34	**0.019**	0.03	0.00 – 0.06	2.14	**0.032**
focus.e * agent.f1	-0.01	-0.04 – 0.03	-0.33	0.740	0.03	0.00 – 0.07	2.08	**0.038**
focus.e * agent.f2	0.00	-0.03 – 0.03	0.24	0.810	0.00	-0.03 – 0.03	0.19	0.852
result.e * focus.e * agent.f1	-0.00	-0.08 – 0.07	-0.11	0.915	-0.01	-0.09 – 0.06	-0.33	0.741
result.e * focus.e * agent.f2	-0.04	-0.10 – 0.01	-1.53	0.127	0.03	-0.03 – 0.08	1.04	0.298

Table S19. Model summaries of LMEMs of ratingBad and ratingGood for experiment 3 and 4 where participants believed they played against a human. Estimated model of ratingBad (left) and ratingGood (right) fixed effects with 95% confidence intervals (CI) of experiments 3 and 4 when people thought they played against robots. The effects included result (win, lose) and focus (ingroup, outgroup) and agent (Cozmo, NAO, human). Agent.f1 compared the Cozmo with the NAO, while agent.f2 compared the NAO with the human. The p-value indicated if the fixed effect had a significant effect on the ratings.

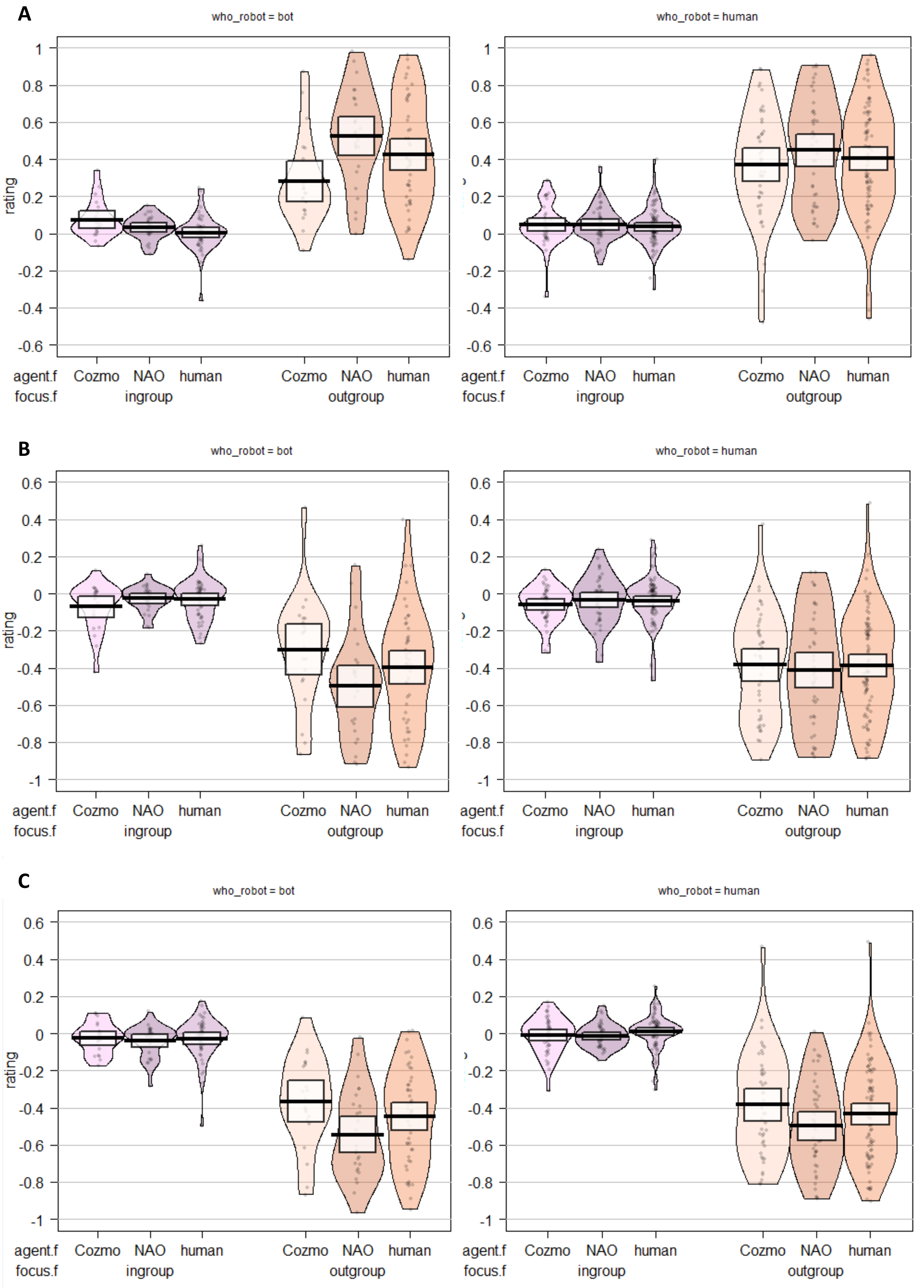

Figure S12. Trial-by-trial ratings of positive and negative reactions to every game outcome for each player divided by whether they believed they played against a bot or a human. An attenuated schadenfreude bias towards the machine-like robot (i.e., Cozmo), but increased schadenfreude bias towards the human-like robot (i.e., NAO) was observed when compared to human players (in the outgroup). On the other hand, the machine-like ingroup robot (i.e., Cozmo) elicited more schadenfreude than either the ingroup NAO or an ingroup human player (A). A decreased empathy bias was observed towards the machine-like robot (i.e., Cozmo), but increased empathy towards the human-

like robot (i.e., NAO) when compared to humans when in the outgroup (B for feeling good when someone won and C for feeling bad when someone lost). There was no difference observed in empathy based on the human likeness of the agents within the ingroup.

References

Arslan, R. C., Walther, M. P., & **Tata, C. S.** (2020). formr: A study framework allowing for automated feedback generation and complex longitudinal experience-sampling studies using R. *Behavior Research Methods*, *52*(1), 376–387. DOI: *https://doi.org/10.3758/s13428-019-01236-y*

Hoorens, V. (1995). Self-Favoring Biases, Self-Presentation, and the Self-Other Asymmetry in Social Comparison. *Journal of Personality*, *63*(4), 793–817. DOI: *https://doi.org/10.1111/j.1467-6494.1995.tb00317.x*

Humphreys, G. W., & **Sui, J.** (2015). The salient self: Social saliency effects based on self-bias. *Journal of Cognitive Psychology*, *27*(2), 129–140. DOI: *https://doi.org/10.1080/20445911.2014.996156*

Lindeman, M. (1997). Ingroup bias, self-enhancement and group identification. *European Journal of Social Psychology*, *27*(3), 337–355. DOI: *https://doi.org/10.1002/(SICI)1099-0992(199705)27:3<337::AID-EJSP825>3.0.CO;2-S*
